# Supplementary material for: Cryptic biodiversity of freshwater fish species in Bangladesh
Source: PLoS One. 2025 Apr 28;20(4):e0318982. doi: 10.1371/journal.pone.0318982 (PMC12036941; doi:10.1371/journal.pone.0318982)
Supplement: Attachment — Submitted filename: Esmaeili et al. 2020_Aphanius barcoding_plosone.pdf [file pone.0318982.s006.pdf]

PLOS ONE

RESEARCH ARTICLE

DNA barcoding and species delimitation of the Old World tooth-carps, family Aphaniidae Hoedeman, 1949 (Teleostei: Cyprinodontiformes)

Hamid Reza Esmaeili[1]*, Azad Teimori[2], Fatah Zarei[1], Golnaz Sayyadzadeh[1]

1 Ichthyology and Molecular Systematics Research Laboratory, Zoology Section, Department of Biology, College of Sciences, Shiraz University, Shiraz, Iran, **2** Department of Biology, Faculty of Sciences, Shahid Bahonar University of Kerman, Kerman, Iran

* hresmaeili@shirazu.ac.ir

Check for updates

OPEN ACCESS

Citation: Esmaeili HR, Teimori A, Zarei F, Sayyadzadeh G (2020) DNA barcoding and species delimitation of the Old World tooth-carps, family Aphaniidae Hoedeman, 1949 (Teleostei: Cyprinodontiformes). PLoS ONE 15(4): e0231717. https://doi.org/10.1371/journal.pone.0231717

Editor: Roberta Cimmaruta, Universita degli Studi della Tuscia, ITALY

Received: December 18, 2019

Accepted: March 30, 2020

Published: April 16, 2020

Peer Review History: PLOS recognizes the benefits of transparency in the peer review process; therefore, we enable the publication of all of the content of peer review and author responses alongside final, published articles. The editorial history of this article is available here: https://doi.org/10.1371/journal.pone.0231717

Copyright: © 2020 Esmaeili et al. This is an open access article distributed under the terms of the Creative Commons Attribution License, which permits unrestricted use, distribution, and reproduction in any medium, provided the original author and source are credited.

Data Availability Statement: All DNA sequences files are available from the Genbank database. Accession numbers are provided in Table 1. Specimens studied are available and can be

Abstract

The fishes, which have currently named *Aphanius* Nardo, 1827 are the relict of the ancient ichthyofauna of the Tethys Sea. For a long time since 1827, the genus name has been subjected to revision by several researchers using mainly morphological features. Until recently, no comprehensive single- or multi-locus DNA barcoding study has been conducted on whole members of the family Aphaniidae. In the present study, by applying four conceptually different molecular species delimitation methods, including one distance-based method, one network-based and two topology-based methods, we examined a single-locus DNA barcode library (COI) diversity for the 268 sequences within the family Aphaniidae from the Old World (57 sequences are new in the present study and 211 sequences were retrieved from NCBI database). The molecular analyses revealed a clearer picture of intra-family relationships and allowed us to clarify the generic names, and also describe a new genus for the family Aphaniidae. Results supported distinction of three major clades related to three genera within this family: i) the first clade includes the *A. mento* group which are placed in a new genus, *Paraphanius* gen. nov., found in the Orontes (= Asi) and Tigris-Euphrates River drainage, the Levant in coastal waters and the Dead Sea basin, western Jordan, and in southern Turkey in the Mediterranean basins as well as in central Turkey. This clade positioned at the base of the phylogenetic tree, (ii) the second clade contains the *A. dispar*-like brackish water tooth-carps which are transferred to the genus *Aphaniops* Hoedeman, 1951 (type species, *Lebias dispar*), distributed in the coastal waters around the Red Sea and the Persian Gulf basins; and (iii) the third clade, the genus *Aphanius* Nardo, 1827 (type species *Aphanius nanus* = *A. fasciatus*) contains all the inland and inland-related tooth-carps, which are mainly distributed in the inland waters in Turkey and Iran and also in the inland-related drainages around the Mediterranean basin.

accessed (deposited in the museums mentioned in the paper).

Funding: The research work was funded by Shiraz University provided to the first Author.

Competing interests: The authors have declared that no competing interests exist.

Introduction

Morphological characters have long been used to describe and identify fish species. Nevertheless, it is known that traditional methods based on morphological properties often do not allow the discovery of cryptic species, frequently leading to taxon misidentification, instability in the number of species and an insufficient indication of the loss of genetic diversity. Since all fields of biology depend on the convenient species identification [1, 2], the species delimitation needs to be efficient and reliable.

DNA barcoding based on the partial nucleotide sequences of the cytochrome c oxidase I (COI) gene has been increasingly used as one of the principal approaches in the bio-identification system of animals [3]. Almost all species can be distinguished by their COI sequences only if the average intraspecific and interspecific genetic distances are significantly different (barcoding gap) [4, 5].

The most important benefits of DNA barcoding are its accuracy to distinguish closely related species, identifying individuals at different developmental stages, discovering cryptic species and to identify the possible causes of synonymies. Therefore, over recent decades, this technology has had a principal contribution to the reliable identification of species [6, 7].

The Old World Cyprinodontiformes (now in the genus *Aphanius* Nardo, 1827), including the extant and fossil species, are widely distributed along the late-period Tethys Sea coastlines. It is known that differences in the Mediterranean Sea level created by glacial and interglacial conditions have largely affected their present-day distribution. Since the tooth-carp fishes are not highly diverse in external morphology, therefore, primary morphological attempts were not quite successful for species identification. For this reason, the systematics of these fishes has been the subject of many studies since the first description of the *Aphanius* species in 1821 by Valenciennes.

What is *Aphanius* Nardo, 1827?

The fishes that have currently named *Aphanius* Nardo, 1827 (ref. 1827a, pp. 34, 39–40; also published in 1827b, col. 487), are the relict of the ancient ichthyofauna of the Tethys Sea. These fishes first appeared in the middle to late Aquitanian. The oldest records, described based on the otolith morphology are from Germany (the Mainz Basin) and SW France (the Aquitaine Basin), 'genus Cyprinodontidarum' [= *Aphanius*] *angulosus* Steurbaut, 1978) (cf. Reichenbacher 2000). *Aphanius princeps*, found in the deposits of the Burdigalian age in Catalonia, NE Spain, is the taxon with the oldest fossil skeleton [8].

The genus *Aphanius* was the only native representative of the family Aphaniidae in the Old World formerly considered to be in the family Cyprinodontidae [9, 10–12]. It was Sethi [13] who for the first time, advocated placing *Aphanius* in a distinct family, Aphaniidae, however, this suggestion did not find general acceptance. Typically, the name *Aphanius* refers to a genus of fresh and brackish water tooth-carps with a wide distribution, basically peri-Mediterranean, extending from the Mediterranean basin including Portugal and Morocco to the Persian Gulf and its eastern regions, including Pakistan and India [9, 14–18]. These fishes are generally small (up to 70 mm long) thriving in coastal and brackish water environments, the inland and land-locked water systems such as spring-stream systems, hot sulphuric springs, and rarely wetlands [16, 19–23].

Systematics and nomenclature history. Since 1827, the genus name has been subjected to revision by several researchers (see Kottelat and Wheeler [24]) and it has been widely used since at least 1926. Originally, the genus was established with two nominal species *Aphanius nanus* Nardo, 1827 and *Aphanius fasciatus* Nardo, 1827 from the Mediterranean waters. Later *A. nanus* was selected as a junior synonym of *Lebias fasciata* Valenciennes in Cuvier &

Valenciennes, 1827 (Jordan [25], p. 121). The type species, therefore, becomes "*fasciatus*", now *A*. *fasciatus* by the subsequent designation of Jordan [25].

Aphanius has been named by some authors as a junior synonym of *Lebias* Goldfuss, 1820 [26]. It should be noted that for more than 150 years *Lebias* had been considered as a junior synonym of *Cyprinodon* Lacépède, 1803. However, with a single exception in 1895, it remained unused since 1846. In addition, Hoedeman [27] has examined four specimens of the genus *Tellia* from the collection in the Paris Museum and proposed that *Tellia* should not be separated from *Aphanius*, while *Aphanius dispar* is quite well separable from *Aphanius*. Therefore, a new generic name *Aphaniops* ("*Aphaniops* = *Aphanius*-opsis; resembling *Aphanius*", anticipated by Hoedeman [27]) was proposed for *A*. *dispar*. The new genus *Aphaniops* Hoedeman, 1951 was based on the following characters; having no dermal sheath around the anterior anal-fin rays, presence of 8–9 dorsal-fin rays in *Aphaniops* versus 10–14 dorsal-fin rays in *Aphanius*, 7–8 pelvic-fin rays in *Aphaniops* in contrast to 5–7 pelvic-fin rays in *Aphanius*. Accordingly, he provided the following key for the discrimination of *Aphanius* from *Aphaniops*:

1. "Dorsal fin with 8–9 rays, ventral fins well developed with 7–8 rays, no dermal sheath around the anterior anal rays, and length up to 80 mm. Genus *Aphaniops*.
2. Dorsal fin with 10–14 rays; ventral fins well developed, rudimentary or entirely wanting, with 5–7 rays if present, the presence of the dermal sheath around first few anal rays, naked or covered with scales, and length up to 65 mm. Genus *Aphanius*".

Also, according to Parenti [28], *Aphanius mento* possesses several diagnostic features not found in other members of the genus *Aphanius*, including a cartilaginous interhyal (ossified in other *Aphanius* species), an embedded urohyal (not embedded in other *Aphanius*), an upturned lower jaw (not upturned in other *Aphanius* species), and a distinctive neuromast pattern on the dorsal surface of the head (less prominently developed in other *Aphanius* species). As a result, she interpreted *A*. *mento* as the derived member of the *Aphanius* clade that should be designated as "*Aphanius*". This taxonomic separation corresponds to the observation of the sulcus morphology of sagittal otolith in the study of Reichenbacher et al. [29]. In the following, the list of synonyms is provided for the genus *Aphanius*:

Aphanius Nardo, *1827*. Synonym of *Lebias* Goldfuss 1820, according to Lazara [30, 26]. Valid as *Aphanius* Nardo 1827, according to Tortonese [31], Parenti [28], Wildekamp et al. [32], Coad [33], Kruppand Schneider [34], Wildekamp [16], Poll & Gosse [35], Seegers [36], Wildekamp et al. [23], Doadrio et al. [37], Huber [38], Blanco et al. [39], Hrbek et al. [40], Kottelat et al. [41], Kottelat & Freyhof [42], Hertwig [43], Coad [44], Teimori et al. [45], Esmaeili et al. [46], Teimori et al. [47], Esmaeili et al. [19], Gholami et al. [48], Keivany & Esmaeili [49], Esmaeili et al. [50], Teimori et al. [51], Pfleiderer et al. [52], Huber [53], Jouladeh-Roudbar et al. [54], Esmaeili et al. [55], Freyhof et al. [9, 10], Çiçek et al. [56], Esmaeili et al. [57], Golani & Fricke [58], Teimori et al. [15, 59], Yoğurtçuoğlu & Freyhof [60], Teimori & Motamedi [61], and Motamedi et al. [62].

Anatolichthys Kosswig & Sözer, *1945*. Valid subgenus, according to Lazara [30].

Anatolichthys splendens Kosswig & Sözer, 1945.

Synonym of *Kosswigichthys* Sözer, 1942, according to Parenti [28]. Synonym of *Aphanius* Nardo, 1827, according to Wildekamp [16], Wildekamp et al. [23], a synonym of *Lebias* Goldfuss, 1820.

Aphaniops Hoedeman, *1951*. Valid subgenus, according to Lazara [30].

Lebias dispar Rüppell, 1829.

Synonym of *Lebias* Goldfuss 1820. Synonym of *Aphanius* Nardo, 1827, according to Tortonese [31], Parenti [28], Wildekamp et al. [32], Krupp & Schneider [34], Wildekamp [16], and Wildekamp et al. [23].

Kosswigichthys Sözer, *1942*. Valid subgenus, according to Lazara [30].

Kosswigichthys asquamatus Sözer,1942.

Synonym of *Lebias* Goldfuss 1820. Synonym of *Aphanius* Nardo, 1827, according to Wildekamp [16] and Wildekamp et al. [23].

Lebias Goldfuss, *1820*. *Lebias banded* Valenciennes, 1821. Valid as *Lebias* Goldfuss, 1820, according to Lazara [26], Costa [63] and Lazara [30]. The invalid generic name, according to Huber [38].

Synonym of *Aphanius* Nardo, 1827, according to Parenti [28].

Micromugil Gulia, *1861*. Valid subgenus, according to Lazara [30].

Synonym of *Aphanius* Nardo, 1827, according to Tortonese [31], Parenti [28], Wildekamp et al. [32], Wildekamp [16] and Wildekamp et al. [23]. Synonym of *Lebias* Goldfuss, 1820.

Tellia Gervais, *1853*. Valid subgenus, according to Huber [38].

Synonym of *Aphanius* Nardo, 1827, according to Parenti [28], Wildekamp et al. [32], Wildekamp [16] and Wildekamp et al. [23]. Synonym of *Lebias* Goldfuss, 1820.

Turkichthys Ermin, *1946*. Valid subgenus, according to Lazara [30].

Turkichthys transgrediens Ermin, 1946. Synonym of *Aphanius* Nardo, 1827, according to Wildekamp [16] and Wildekamp et al. [23]. Synonym of *Lebias* Goldfuss, 1820.

This background leads to the question: is *Aphanius* a monophyletic group? Parenti [28] has implied the paraphyly of *Aphanius* (the genus is probably not monophyletic). In addition, Nelson et al. [64] noted that the genus *Aphanius* probably is not a monophyletic group.

To date, 44 species have been described under the genus *Aphanius* [65]. The Near East, especially Iran and Turkey host the largest number of species [11, 16, 40, 44, 66]. As mentioned above, numerous authors have so far studied different aspects of these fishes, including the phenotypic variation, embryology, allopatric distribution, species diversity, historical zoogeography, evolutionary history and phylogenetic relationships [11, 23, 28, 67–79]. Particularly, significant progress has been made during the past decade to reconstruct the phylogenetic relationships of *Aphanius* species in the Near East [10, 15, 19–22, 40, 44, 46–48, 50, 51, 80–82]. Nevertheless, no comprehensive single- or multi-locus DNA barcoding study has been conducted for species delimitation in the family Aphaniidae.

In this study, by applying four conceptually different molecular species delimitation methods including one distance-based method, one network-based and two topology-based methods, we examined a single-locus DNA barcode library (COI) diversity for 268 sequences within the family Aphaniidae from the Old World (57 sequences are new in this study and 211 sequences retrieved from NCBI database). The aims of our study were (i) to use DNA barcoding as a tool for species delimitation within the family Aphaniidae, ii) to give a clearer picture of intra-family relationships and (iii) to clarify the generic names of the species group within the family. The DNA barcode records generated in this study are discussed by considering some morphological features of these fishes.

Materials and methods

Ethics statement

All fish species were caught in the inland water bodies (not national parks, other protected areas, or private areas, etc.), so no specific permissions were required for these locations/activities. Ethical approval was given by the Bioethics Committee of Biology Department, Shiraz University. Specimen collection and maintenance were performed in strict accordance

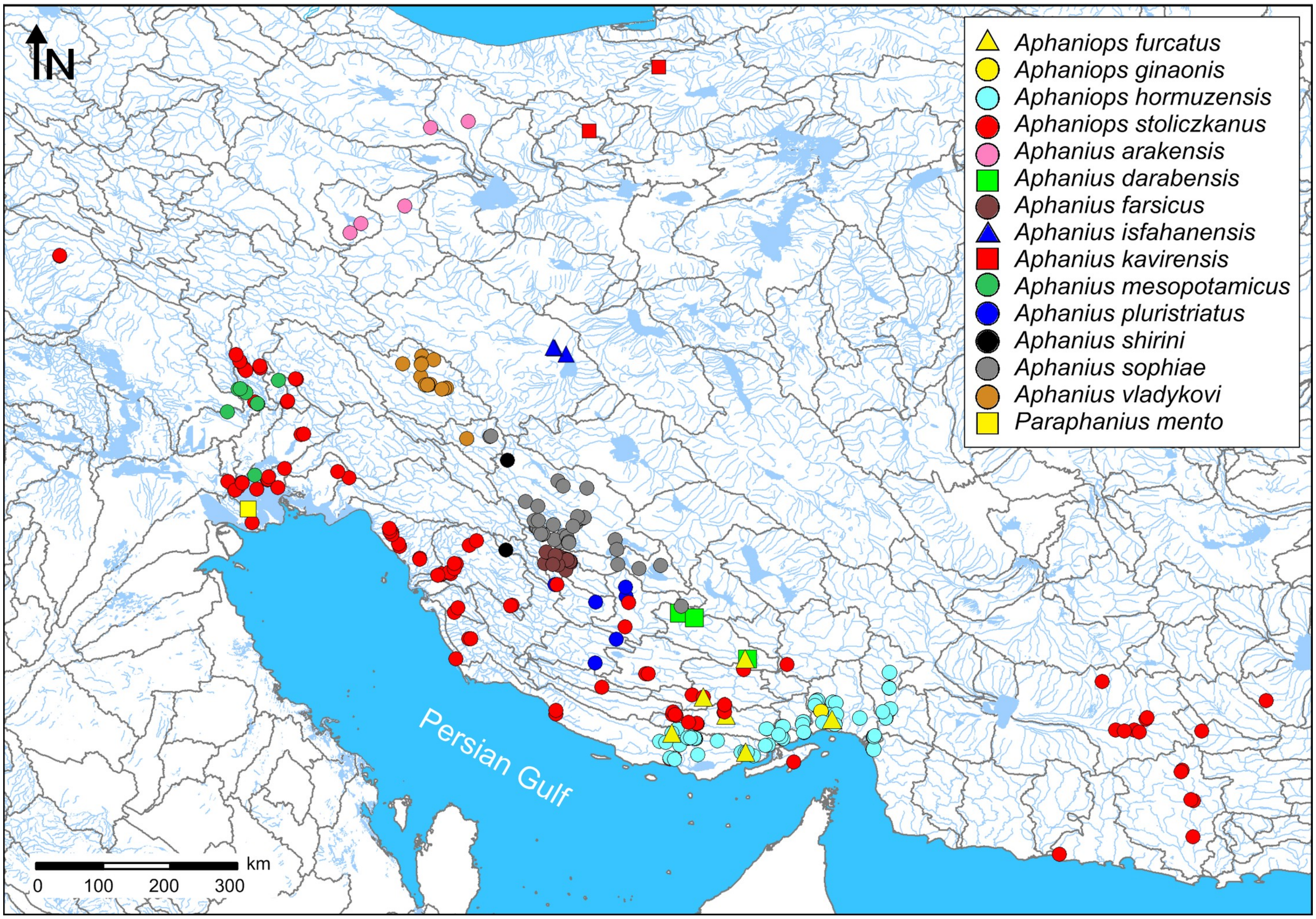

Fig 1. Overview of the distribution areas and the sampling sites of the tooth-carps from Iran. The map was originally designed in DIVA-GIS ver. 7.5 (https://www.diva-gis.org/).

https://doi.org/10.1371/journal.pone.0231717.g001

with the recommendations of the Bioethics Committee of Biology Department, Shiraz University.

Sampling and external morphological identification

A total of 57 specimens belonging to 14 species were sampled from various localities in Iran by using a hand net (Fig 1, Table 1). After anesthesia with 1% clove oil solution, these specimens were kept in 96% ethanol for molecular studies. The fish individuals were identified in the laboratory by two authors (H.R. Esmaeili and A. Teimori) based on the external morphology and including coloration, their geographic distribution and also by considering their original species descriptions and recently published articles [19, 33, 40, 44–48, 51, 83]. Meristic counts were done externally (not using cleared and stained specimens).

DNA extraction, PCR and sequencing

In a sterile condition, small fragments of the dorsal muscle from alcohol-preserved specimens were sampled and placed in 96-well Eppendorf PCR plates. Genomic DNA of 57 specimens

Table 1. New COI sequences for the tooth-carp species sampled from different drainage basins in Iran.

Species	Current proposed status	Basin/locality/coordinates	ZM-CBSU numbers	Accession No.
A. vladykovi	*Aphanius vladykovi*	Karoun River/Gandoman wetland/31° 50'05.0"N 51°04'36.4"E	Ex85C9, Ex85C8, Ex85C10, Ex59G5	MT102153, MT102154, MT102155, MT102156
Aphanius sp.	*Aphanius* sp.	Sirjan/Nahr-e-Masih spring/30°01'56.6"N 54°19'54.5"E	ZG314, ZG313, ZG312, ZG311	MT102157, MT102158, MT102159, MT102160
A. sophiae	*Aphanius sophiae*	Kor River/ Marvdasht/29°52'19.7"N 52° 29'48.4"E	ZG129, ZG128, ZG127	MT102161, MT102162, MT102163
		Karoun River/Bibiseydan spring/31° 11'2.3"N 51°26'59.2"E	ZG327, ZG326, ZG325	MT102164, MT102165, MT102166
		Helleh/ Arjan wetland/29°39'20.99"N 51° 59'14.39"E	ZG112, ZG109, ZG108	MT102167, MT102168, MT102169
A. shirini	*Aphanius shirini*	Kor River/Khosroshirin River/30° 53'29.5"N 52°00'36.8"E	ZG272, ZG270, ZG267	MT102170, MT102171, MT102172
		Helleh/ Arjan wetland/29°39'20.99"N 51° 59'14.39"E	ZG113	MT102173
A. pluristriatus	*Aphanius pluristriatus*	Mond River/Jahrom/28°26'N 53°30'E	Ex90B8, Ex90B6, Ex90B11	MT102174, MT102175, MT102176
		Mond River/ Khonj city/28°06'32"N 53° 12'59"E	ZG120, ZG115, ZG114	MT102204, MT102205, MT102206
A. mesopotamicus	*Aphanius mesopotamicus*	Karoun River/ Jarahi River/30°41'01.4"N 48°32'22.7"E	ZG320, ZG319, ZG317, ZG315	MT102177, MT102178, MT102179, MT102180
A. kavirensis	*Aphanius kavirensis*	Kavir/ Cheshmeh-e-Ali spring/36° 16'45.45.6"N 54°05'01.6"E	ZG141, ZG130, ZG1291, ZG1281	MT102181, MT102182, MT102183, MT102184
A. isfahanensis	*Aphanius isfahanensis*	Esfahan/Varzaneh, Zayandehud River/32° 25'26.28"N 52°39'14.3"E	ZG342, ZG341, Ex53H4, Ex53H3	MT102185, MT102186, MT102187, MT102188
A. ginaonis	*Aphaniops ginaonis*	Hormuz/Genow spring/27°26'51.0"N 56° 18'10.0"E	divmxext3	MT102189
A. furcatus	*Aphaniops furcatus*	Hormuz/Kol River/27°38'19.0"N 54° 41'33.2"E	Ex88F2	MT102190
		Hormuz/Khurgu spring/27°31'21.3"N 56° 28'12.7"E	Ex88F1	MT102191
		Hormuz/Faryab spring/27°26'01.0"N 54° 16'43.0"E	Ex88E11	MT102192
A. farsicus	*Aphanius farsicus*	Maharlu Lake basin/Barmeshoor spring/ 29°27'9.51"N 52°42'0.05"E	ZG249, ZG140, ZG139, SExS02F7, SExS02F6, SExS02F5	MT102193, MT102194, MT102195, MT102196, MT102197, MT102198
A. stoliczkanus	*Aphaniops stoliczkanus*	Persian Gulf/ShourAb at Emamzadeh Shahid/28 32 35.3N, 52 21 43.8E	A621F	MT102199
A. darabensis	*Aphanius darabensis*	Hormuz/Korsia-Banaki spring/28°46'25"N 54°23'48"E	ZG344, ZG137, ZG134, A622F	MT102200, MT102201, MT102202, MT102203
A. arakensis	*Aphanius arakensis*	Namak Lake/Arak spring/34°00'35.1"N 49° 50'50.8"E	ZG351, ZG349, ZG348	MT102207, MT102208, MT102209

https://doi.org/10.1371/journal.pone.0231717.t001

belonging to morphologically identified species based on external characters was purified by extraction from tissues in the presence of high concentrations of Guanidinium Thiocyanate and passage of the extracts through a fiberglass membrane (AcroPrep 1 μM glass fiber; PALL 5051) as described by Vargas et al. [84]. The standard DNA barcode region for vertebrates (COI) was amplified using the primer pair named FishF1 (`5'TCAACCAACCACAAAGACAT TGGCAC3'`) and FishR1 (`5'TAGACTTCTGGGTGGCCAAAGAATCA3'`) [4]. The amplification process was performed using Master Mix in a total volume of 25 μl containing 12.5 μl of a Ready 2X PCR Master Mix (Genetbio, Cat. no. G-2000), 0.5 μl of each primer (10 pmol/μl), 5 μl of the DNA template and 6.5 μl dd water. The amplification was performed on a Bioer XP Thermal Cycler (Bioer Technology Co. Ltd., Hangzhou, China), programmed as following: an initial denaturation at 94°C for 3 min, 35 cycles with denaturation at 94°C for 50 s, annealing

at 52˚C for 1min, and a final extension phase at 72˚C for 5 min. After purification with the ExoASP-IT® (usb) kit, the PCR products were Sanger sequenced with BigDye® Terminator v3.1 Cycle Sequencing Kit (Applied Biosystems, Foster City, CA) on an ABI PRISM 3730xl DNA Analyzer (Applied Biosystems, Foster City, CA) by the Macrogen Company of South Korea.

Phylogenetic reconstruction

For obtaining a reliable outcome, a total of 211 COI sequences [10, 67, 85–88] from 28 *Aphanius* species were retrieved from GenBank and included in the phylogenetic analyses (Table 2). The goby killifish, *Aphyosemion franzwerneri* (Accession number: EF417044) was used as an outgroup. The BioEdit ver. 7.0.4 [89] was used to read the DNA chromatograms. The mtDNA sequences were first aligned with the ClustalW procedure implemented in MEGA ver. 7[90], and then were aligned manually. New sequences are deposited in NCBI Genbank (www.ncbi.nlm.nih.gov) under accession numbers MT102153-MT102209. We examined the substitution saturation with DAMBE ver. 7.2.7 [91]. The nucleotide substitution model best fitting the COI barcode library for the genus *Aphanius* was obtained using Akaike and Bayesian information criteria (AIC & BIC), and a decision theory method (DT) in JModelTest ver. 2.1.3 [92]. All criteria suggested GTR+I+G as the best-fit model for the sequence set. For phylogenetic reconstruction, the Bayesian method was run based on four simultaneous runs of four Markov chains for 100,000,000 generations and a burn-in of 15% of the initial trees in MrBayes ver. 3.2.6 [93]. An ultrametric gene tree was generated using the uncorrelated lognormal relaxed clock model [94] and the birth-death model in BEAST ver.1.8.2 [95]. BEAUTi ver. 1.8.2 [95] was used to implement the run settings: 100 million chain length, sampling each 3000th tree, the uncorrelated lognormal relaxed clock, birth-death model and GTR+I+G substitution model with four gamma categories. Tracer ver. 1.6 [96] was used to test the appropriateness of parameters. The maximum age for *Aphanius* was set at 34 Ma based on the oldest known fossil of the Old World killifishes, *Prolebias stenoura* Sauvage, 1874 [97]. Results were visualized in Tree Annotator ver. 1.8.2[98] with a 10% burn-in rate, 95% highest posterior density of divergence times and under the maximum clade credibility option for the consensus tree. In addition, we generated a Maximum Likelihood (ML) tree with 3,000 bootstrap replicates using RAxML ver. 7.2.5 [99] under the GTR+I+G nucleotide substitution model, with fast bootstrap.

Molecular species delimitation

Four molecular species delimitation methods were applied to reduce difficulties when using only single parameter estimates and to compare results of conceptually different approaches, including a distance-based method, a network-based, and two topology-based approaches. The Automatic Barcode Gap Discovery (ABGD) method [100] uses the distribution of pairwise differences to detect a barcode gap dividing hypothetical species in the sequence set by assuring that intraspecific and interspecific distances do not overlap. We tested a set of COI sequence of the studied tooth-carps on the ABGD web server (https://bioinfo.mnhn.fr/abi/public/abgd/) with a combination of ABGD settings within the parameter range of Pmin = 0.001, Pmax = 0.1 and gap width = 0.1–1.2, all for a total of 20 steps and applying a Kimura-2-parameter (K2P) [101] corrected genetic distance matrix calculated using MEGA ver. 7.0 [90]. The reversed Statistical Parsimony (SP) method [102] is a network-based system that delineates hypothetical species using the network topology. The TCS ver. 1.21 [103] software was used to calculate a statistical parsimony network. The haplotypes are clustered into two separate networks or species if the number of mutations between two neighboring haplotypes, being more than the connection probability. We used a 95% connection probability

Table 2. Details of 211 COI sequences belonging to 28 *Aphanius* species acquired from the GenBank database.

Species	N[a]	GenBank accession numbers						
A. alexandri	3	KJ552602	KJ552715	KJ552647				
A. almiriensis	8	KJ552360	KJ552520	KJ552640	KJ552735	MH410024	MH410025	MH410026
		MH410027						
A. anatoliae	10	AY356565	KJ552467	KJ552604	KJ552353	KJ552362	KJ552421	KJ552426
		KJ552691	KJ552704	KJ552628				
A. apodus	3	KJ552553	KJ552606	KJ552719				
A. baeticus	4	KJ552741	KJ552418	KJ552475	KJ552714			
A. dispar	32	MF918578	MG013854	MG013855	MG013857	MF918579	MF918580	MG013846
		MG013847	MG013848	MG013849	MG013850	MG013852	MG013853	MG013859
		MG013860	MG013861	MG013862	MG013863	MF918576	MF918577	MG013864
		MG013865	MG013866	MG013867	MF918581	MF918582	MF918583	MG013874
		MG013873	MG013875	MF918588	MF918589			
A. fasciatus	32	KJ552453	KJ552516	MH410031	MH410032	KJ552372	KJ552597	KJ552687
		KJ552699	MH492715	KJ552449	KJ552618	KJ552621	KJ552624	KJ552744
		MH492716	MH492717	MH492718	MH492719	MH492720	KJ552363	KJ552667
		KJ552751	KJ552757	KJ552428	KJ552559	KJ552584	KJ552610	KJ552726
		KJ552524	MH410028	MH410029	MH410030			
A. fontinalis	4	KJ552742	KJ552700	KJ552560	KJ552722			
A. ginaonis	3	MF918590	MF918591	MF918592				
A. iberus	7	KJ552361	KJ552729	KJ552419	KJ552617	KJ552466	KJ552653	KJ552705
A. iconii	2	KJ552481	KJ552688					
A. kruppi	5	MF918593	MF918594	MF918595	MF918596	MF918598		
A. maeandricus	5	KJ552515	KJ552532	KJ552605	KJ552543	KJ552645		
A. mento	9	KJ552373	KJ552492	KJ552576	KJ552442	KJ552750	KJ552446	KJ552673
		KJ552713	KJ552409					
A. mentoides	5	KJ552397	KJ552456	KJ552482	KJ552668	KJ552702		
A. meridionalis	7	KJ552432	KJ552461	KJ552533	KJ552544	KJ552660	KJ552706	KJ552743
A. orontis	2	KJ552423	KJ552683					
A. richardsoni	8	KJ552395	KJ552571	KJ552638	KJ552643	KJ552736	MF918599	MF918600
		MF918601						
A. saldae	2	KJ552398	KJ552630					
A. saourensis	5	KJ552386	KJ552497	KJ552623	KJ552670	KJ552701		
A. similis	4	KJ552414	KJ552500	KJ552367	KJ552393			
A. sirhani	3	MF918602	MF918603	KJ552402				
A. stiassnyae	5	MG013868	MG013869	MG013870	MG013871	MG013872		
A. stoliczkanus	23	MF918615	MF918616	MF918611	MF918612	MF918613	MF918614	MF918617
		MF918618	MF918619	MF918620	MF918621	MF918622	MF918605	MF918606
		MF918607	MF918608	MF918609	MF918624	KU499803	KU499804	KJ552389
		KJ552732	MF918622					
A. sureyanus	2	KJ552468	KJ552758					
A. transgrediens	8	KJ552368	KJ552404	KJ552410	KJ552452	KJ552528	KJ552574	KJ552679
		KJ552710						
A. villwocki	2	KJ552538	KJ552582					
Aphanius sp. (Syria)	8	KJ552593	KJ552483	KJ552542	KJ552692	KJ552471	KJ552536	KJ552635
		KJ552711						

[a]Number of sequences

https://doi.org/10.1371/journal.pone.0231717.t002

threshold to delineate hypothetical species. The Bayesian Poisson Tree Process (bPTP) approach [104] uses a tree topology to delineate species. It assumes that each mutation event has a non-null probability of forming a new species and, thus, the number of interspecific mutations is significantly higher than the number of intraspecific mutations. The bPTP server (https://species.h-its.org/ptp/) was used with a Bayesian topology produced in MrBayes ver. 3.2.6 as input tree. The analysis was run under default settings. The Bayesian General Mixed Yule-Coalescent (bGMYC) method [105] is conceptually similar to bPTP and uses a tree topology to infer species hypotheses, but unlike bPTP, it applies an ultrametric tree as an input file. This method implies that each node of a gene tree corresponds to one of these two possible events: (i) divergence between species after a strict Yule process [106]; or (ii) neutral coalescent events of lineages forming a species [107]. Since the rate of coalescent events is higher than speciation, it is possible to define a limit on a phylogenetic tree between interspecific and intraspecific divergence, delimiting clusters of specimens, which are genetically isolated, and independently evolving lineages or species. This analysis was performed using the bGMYC package [105] for R ver. 3.6.1 [108]on an ultrametric tree produced in BEAST ver. 1.8.2 as an input file.

Results

Molecular species delimitation

The final COI alignment included 268 sequences and one out-group sequence (i.e., *Aphyosemion franzwerneri*) trimmed to 609 bps. The nucleotide substitution pattern showed that the sequences have not reached substitution saturation and are therefore well applicable for phylogenetic analyses. The data were analyzed using the Bayesian Inference and Maximum Likelihood (ML) methods (Fig 2). All the nominal species were recovered as monophyletic except *A. dispar* (Rüppell, 1829), *A. stiassnyae* Getahun & Lazara, 2001 (from Ethiopia, Lake Afrera), *A. pluristriatus* (Jenkins, 1910), and *A. fontinalis* Aksiray, 1948 (from Turkey). The phylogenetic position of *A. richardsoni* (Boulenger, 1907), *A. stiassnyae*, *A. kruppi*, *A. ginaonis* and *A. stoliczkanus* made *A. dispar* a paraphyletic species. In addition, the phylogenetic position of *A. sureyanus* (Neu, 1937) made *A. fontinalis* paraphyletic. The clade formed by *A. pluristriatus A. kavirensis*, *A. sophiae*, *Aphanius* sp. and *A. mesopotamicus* is plagued with very low levels of interspecific sequence divergence (S1 Table), and low values of posterior probabilities and bootstraps (Fig 2).

The four conceptually different molecular species delimitation methods revealed 35–48 species (Fig 2). The ABGD method delineated 40 species (Fig 2). Out of 40 nominal species, 26 (65%) were delimited through the ABGD tool. It indicated potential species-level diversity in five nominal species: *A. dispar*, *A. kruppi*, *A. ginaonis*, *A. stoliczkanus*, and *A. anatoliae*. ABGD presented the following groups of nominal species/sequences with low genetic diversity): (i) *A. stiassnyae* and *A. dispar* specimens from Lake-Assal (Djibouti), Afrera spring (North Ethiopia), Lake Abaeded (Ethiopia), Gali Colluli River (Eritrea), Zariga River (Eritrea), and Shukaray River (Eritrea; MF918580 and MF918579); (ii) *A. arakensis*, *A. pluristriatus*, *A. kavirensis*, *A. sophiae*, *Aphanius* sp. and *A. mesopotamicus* (all from Iran); and (iii) *A. fontinalis* and *A. sureyanus*.

The SP method delineated 35 species (Fig 2). Out of 40 morphologically identified species, 26 (65%) were delimited nicely through the SP tool. The SP analysis presented potential species-level diversity in two nominal species including *A. dispar* and *A. anatoliae*. This method also revealed low genetic diversity in the COI barcode region of the following species:(i) *A. stiassnyae* and *A. dispar* specimens from Lac-Assal (Djibouti), Afrera spring (North Ethiopia), Lake Abaeded (Ethiopia), Gali Colluli river (Eritrea), Zariga river (Eritrea), and Shukaray river

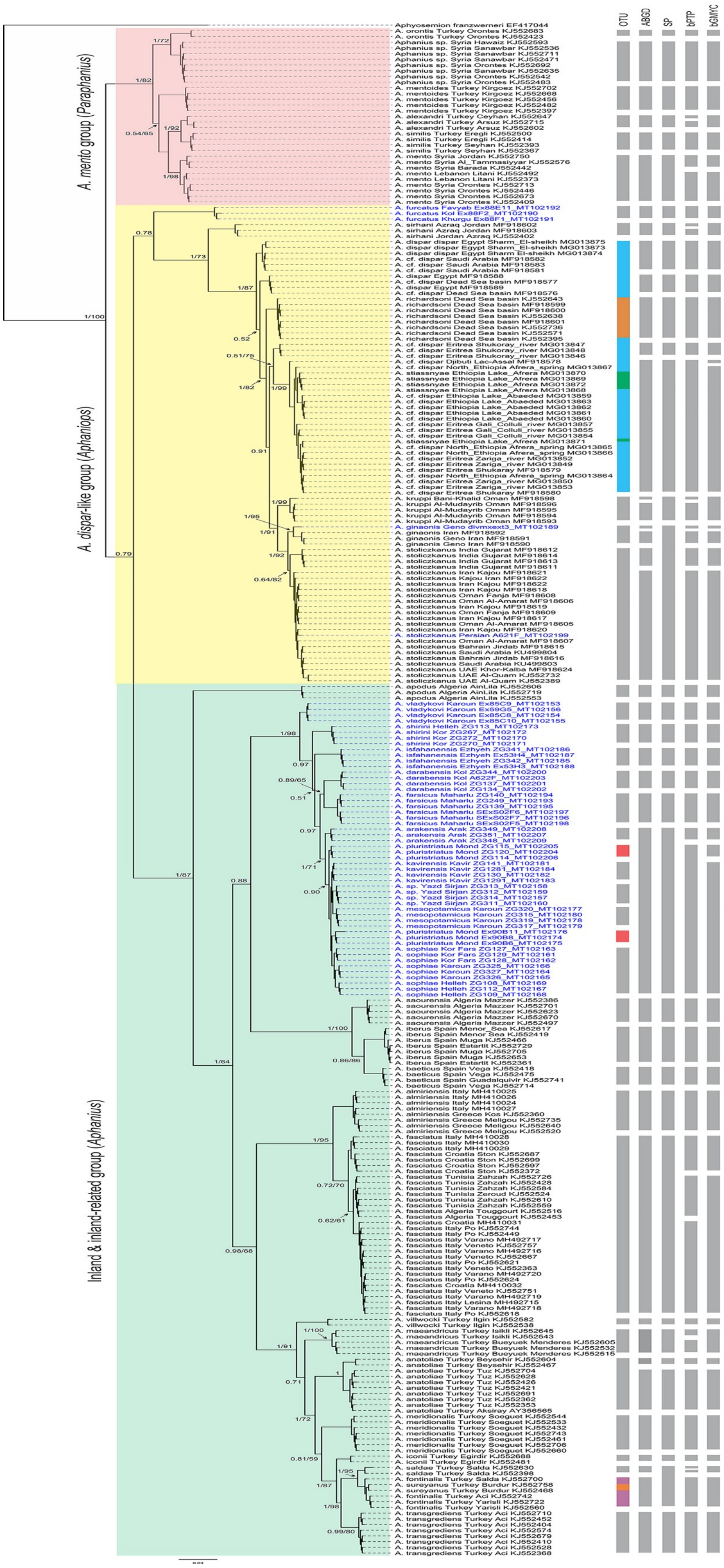
A. mento group (Paraphanius)
A. dispar-like group (Aphaniops)
Inland & inland-related group (Aphanius)
OTU
ABGD
SP
bPTP
bGMYC

Fig 2. Bayesian and Maximum Likelihood phylogeny reconstructed based on COI gene sequences visualizing the results of the four different molecular species delimitation Methods. ABGD, Automatic Barcode Gap Discovery; SP, reversed Statistical Parsimony; bPTP, Bayesian Poisson tree process model; bGMYC, Bayesian General Mixed Yule-Coalescent model. Operational taxonomic unit (OTU) corresponding to the morphological or nominal species. The numbers before and after the slash at important nodes are posterior probability and bootstrap values, respectively (values lower than 0.5 and 50% are not shown at these nodes).

https://doi.org/10.1371/journal.pone.0231717.g002

(Eritrea; MF918580 and MF918579); (ii) *A. ginaonis* and *A. stoliczkanus*; (iii) *A. arakensis*, *A. pluristriatus*, *A. kavirensis*, *A. sophiae*, *Aphanius* sp. and *A. mesopotamicus* (all from Iran); and (iv) *A. saldae*, *A. fontinalis*, and *A. sureyanus*.

The bGMYC method split the dataset into 44 species (Fig 2). Out of 40 nominal species, 26 (65%) were delimited through the bGMYC tool. It presented potential species-level diversity in six nominal species: (i) two potential species in *A. mento*; (ii) five potential species in *A. dispar*; (iii) two potential species in *A. ginaonis*; (iv) two potential species in *A. stoliczkanus*; (v) two potential species in *A. fasciatus*; and (vi) two potential species in *A. anatoliae*. The bGMYC method is similar to bPTP, but it uses an ultrametric tree to delimit species. As no mutation rate has been reported for COI in genus *Aphanius*, the maximum age for *Aphanius* was set at 34 Ma based on the oldest known fossil of the Old World killifishes to produce an ultrametric tree. For this reason, and since credible intervals for some species-group nodes were broad (not shown), the bGMYC results should be cautionary interpreted.

Finally, the delimitation method based on bPTP yielded the largest number of species (48 species; Fig 2). Out of 40 nominal species, 21 (52.5%) were delimited through the bPTP tool. It indicated potential species-level diversity in 11 nominal species: (i) two potential species in *A. alexandri*; (ii) two potential species in *A. mento*; (iii) two potential species in *A. sirhani*; (iv) four potential species in *A. dispar*; (v) two potential species in *A. kruppi*; (vi) two potential species in *A. ginaonis*; (vii) two potential species in *A. stoliczkanus*; (viii) three potential species in *A. fasciatus*; (ix) two potential species in *A. maeandricus*; (x) two potential species in *A. anatoliae*; (xi) two potential species in *A. saldae*. This method considered low genetic distance in the COI barcode region for the following groups: (i) *A. stiassnyae* and *A. dispar* specimens from Lac-Assal (Djibouti), Afrera spring (North Ethiopia), Lake Abaeded (Ethiopia), Gali Colluli river (Eritrea), Zariga river (Eritrea), and Shukaray river (Eritrea; including MF918580 and MF918579); (ii) *A. pluristriatus*, *A. kavirensis*, *A. sophiae*, *Aphanius* sp. from Yazd and *A. mesopotamicus*; and (iii) *A. fontinalis* and *A. sureyanus*.

Overall, 28 of 35–48 putative species delimited by the four molecular species delimitation approaches could be morphologically linked to a species name by at least one method, as follows (Fig 2): *A. orontis*, *Aphanius* sp. from Syria, *A. mentoides*, *A. alexandri*, *A. similis*, *A. mento*, *A. furcatus*, *A. sirhani*, *A. richardsoni*, *A. kruppi*, *A. apodus*, *A. vladykovi*, *A. shirini*, *A. isfahanensis*, *A. darabensis*, *A. farsicus*, *A. arakensis*, *A. saourensis*, *A. iberus*, *A. baeticus*, *A. almiriensis*, *A. fasciatus*, *A. villwocki*, *A. maeandricus*, *A. meridionalis*, *A. iconii*, *A. saldae* and *A. transgrediens*. Overall, 10 nominal species represented a high level of diversity by at least one of the four molecular species delimitation methods: *A. alexandri*, *A. mento*, *A. sirhani*, *A. dispar*, *A. kruppi*, *A. ginaonis*, *A. stoliczkanus*, *A. fasciatus*, *A. maeandricus*, and *A. anatoliae*.

In this study, the average K2P genetic distance between a group containing *A. mento* versus a group containing brackish water taxa was 17%, while the average K2P genetic distance between a group containing *A. mento* versus a group containing the inland and inland-related taxa was 19.6% (Table 3). The mean K2P genetic distances within the brackish water group, within the inland and inland-related group, and within the *A. mento* group were 3.7% (S.E. = 0.4%), 13.1% (S.E. = 1%) and 4.8% (S.E. = 0.5%), respectively (Table 4).

Table 3. Mean K2P distances (%) between three groups of the Old World tooth-carps.

	Inland and inland-related group (*Aphanius*)	*A. dispar*-like group (*Aphaniops*)	*A. mento* group (*Paraphanius*)
Inland and inland-related group (*Aphanius*)	0		
A. dispar-like group (*Aphaniops*)	19.7	0	
A. mento group (*Paraphanius*)	19.6	17.0	0

https://doi.org/10.1371/journal.pone.0231717.t003

Paraphanius gen. nov. Esmaeili' Teimori, Zarei & Sayyadzadeh

(Fig 3)

Diagnosis. *Paraphanius* can be distinguished from the other genera of Aphaniidae, *Aphanius* (Fig 4) and *Aphaniops* (Fig 5) by considering the following characteristics: unique colour pattern of breeding males being dark blue-black to dark brown or almost black with iridescent blue-white to silvery spots regularly-arranged on the fins as curved lines, and irregularly on the body (sometimes as irregular vertical bars and sometimes the spots are vertically elongate), the absence of the wide and black bars on the caudal fin of the male (vs. the presence of 3 wide black bars on the caudal fin of the male in *Aphaniops*), the absence of vertical bands on the body of the male, (the presence of vertical bars in the male of *Aphanius*), a higher number of dorsal fin rays than in *Aphaniops* (9–14 in *Paraphanius* vs. 8–9 in *Aphaniops*), a cartilaginous interhyal (versus ossified in *Aphanius* and *Aphaniops*), an embedded urohyal in the fold of branchiostegal membranes (versus not embedded in *Aphanius* and *Aphaniops*), an upturned lower jaw (versus not upturned in *Aphanius* and *Aphaniops*), and a distinctive neuromast pattern on the dorsal surface of the head (versus less prominently developed in *Aphanius* and *Aphaniops*) based on Parenti [28]. The epural is thin or less developed in *Paraphanius*, while it is thick and well developed in *Aphanius* and *Aphaniops*. Similar to *Aphanius*, the otolith in *Paraphanius* has a straight sulcus (versus a sulcus which is distinctly bent terminally in *Aphaniops*).

Remark. It has been proposed that the *Paraphanius* group is more closely related to the Anatolian *Kosswigichthys* Sözer, 1942 and the South American *Orestias* Valenciennes in Cuvier and Valenciennes, 1846 than the genus *Aphanius* (Parenti [28]:524). However, *Kosswigichthy* is considered as a synonym of *Aphanius* Nardo, 1827 by Wildekamp [16]:19. In addition, according to osteological information provided by Parenti [28]:521, Aphaniidae is distinguished from Orestiidae by having a vomer (vs. absent), a cartilaginous mesethmoid (vs. ossified) and an ossified interhyal (not ossified). Based on the tree topology, populations from the Orontes (= Asi) and the Levant in coastal and Dead Sea basins should be considered as distinct taxa.

Based on the tree topology, *A. alexandri*, *A. mento*, *A. mentoides*, *A. orontis*, *A. similis*, and *Aphanius* sp. make a distinct clade sister to *A. dispar* plus *A. fasciatus* group. The validity of the *A. mento* group has been under debate. Akşiray [109] published the description of one new species and 15 new subspecies of *Aphanius* from Turkey. However, Villwock [110, 111] as well

Table 4. Mean of the within group K2P distance (%) for the Old World tooth-carps.

Tooth-carp groups	Mean of the within group K2P distance	S.E.[a]
Inland and inland-related group (*Aphanius*)	13.1	1
A. dispar-like group (*Aphaniops*)	3.7	0.4
A. mento group (*Paraphanius*)	4.8	0.5

[a]standard error

https://doi.org/10.1371/journal.pone.0231717.t004

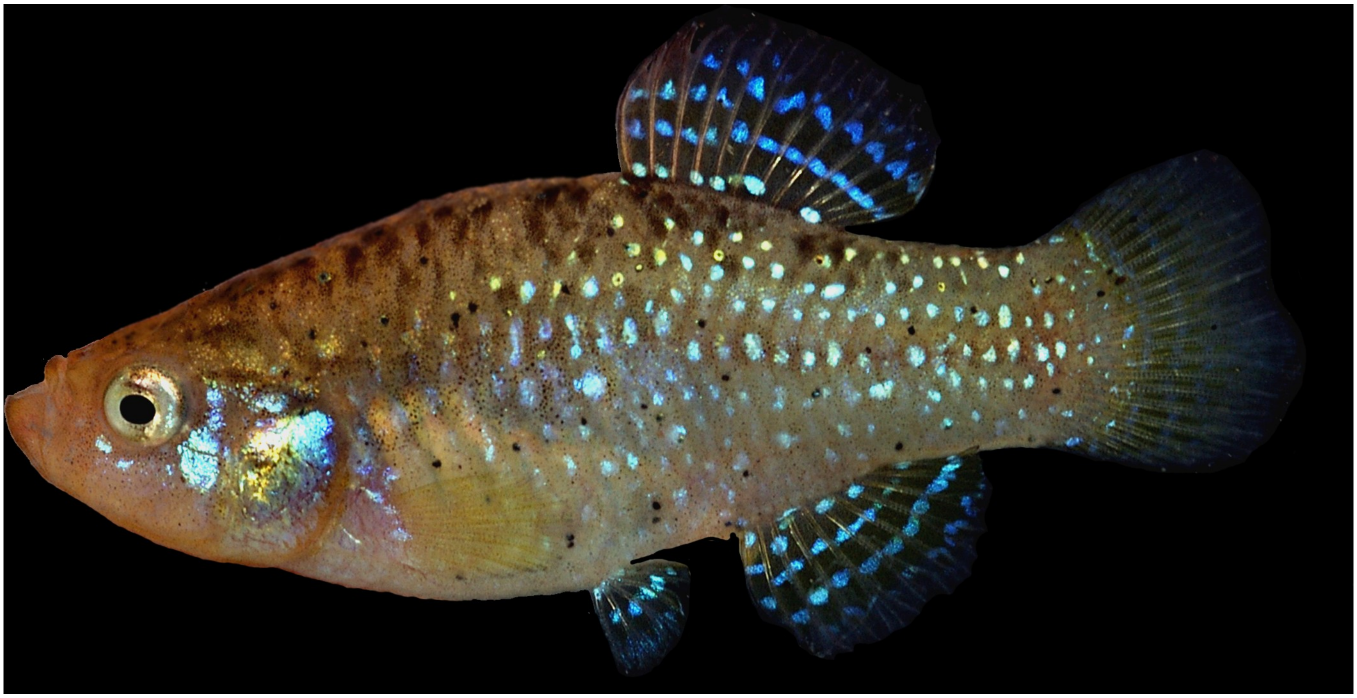

Fig 3. ***Paraphanius mento*, male, Spring of Barada (provided by J. Freyhof).**

https://doi.org/10.1371/journal.pone.0231717.g003

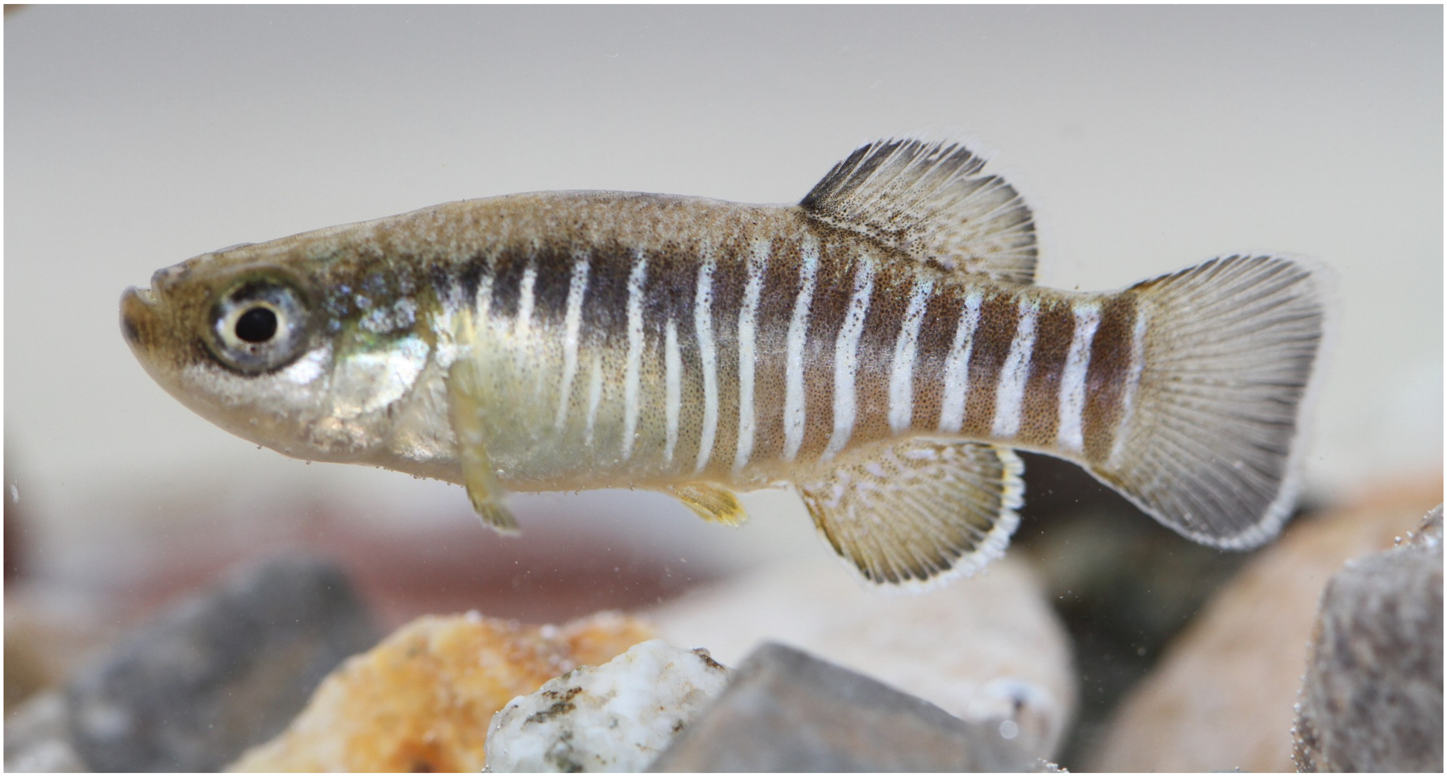

Fig 4. ***Aphanius pluristriatus*, male, Mond River drainage, Iran.**

https://doi.org/10.1371/journal.pone.0231717.g004

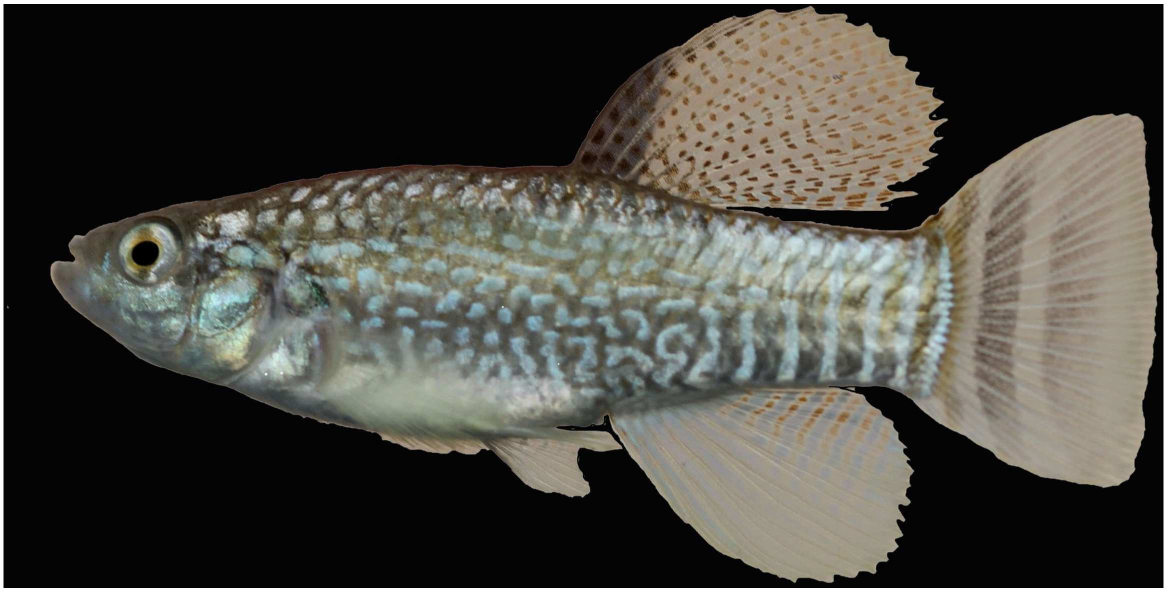

Fig 5. *Aphaniops stoliczkanus*, male, Tigris River drainage, Ilam, Iran.

https://doi.org/10.1371/journal.pone.0231717.g005

as Wildekamp [16] and Wildekamp et al. [23] did not accept any of these 16 taxa as valid. Wildekamp et al. [23] placed *A*. *cypris alexandri*, *A*. *cypris boulengeri*, *A*. *cypris orontis*, *A*. *sophiae similis* and *A*. *sophiae mentoides* in the synonymy of *A*. *mento*. These subspecies have species rank and are considered in the genus *Paraphanius* here.

The species diagnosis is valid also as a genus-level diagnosis.

Type species. *Aphanius mento* (Heckel, 1843), Mossul, northern Iraq (36˚18'N, 43˚18'E). Possible syntypes: NMW 21699–704 (6), 59832 (21), illustrated by Heckel 1843: Pl. 6 (Fig 4).

Etymology. The generic name, *Paraphanius*, is made out of the Greek word Para, meaning "near, resembling", and *Aphanius*, from the Greek word Aphanus, meaning "invisible", a genus of tooth-carp fish, in reference to its phylogenetic position being sister to *Aphanius* and *Aphaniops*.

Found in the Orontes (= Asi) and Tigris-Euphrates basins, the Levant in coastal and the Dead Sea basins, western Jordan, and in southern Turkey in Mediterranean basins as well as in central Turkey.

***Paraphanius mento*, the type species.** Scales along the body 23–28, a total of 9–14 rays in the dorsal fin, a total of 9–13 in the anal fin, 12–16 and 4–6 rays in pectoral and pelvic fins respectively. Total gill rakers 11–15. The jaw teeth display a long middle cusp and short lateral cusps, otoliths are triangular in shape, have a prominent rostrum, and a straight sulcus. A total of 25–26 vertebrae present in the vertebral column, five preural vertebrae (PU1–5) with long haemal and neural spines, the presence of a small plate in front of the spines of PU5, flank scales are squarish to a vertical oval in general shape. There is no pelvic axillary scale. The gut is s-shaped.

Included species. *Aphanius alexandri*, *A*. *mento*, *A*. *mentoides*, *A*. *orontis*, *A*. *similis*, and *Aphanius* sp.

Discussion

Species delimitation

Traditional morphology-based approaches are unable to distinguish cryptic entities and unveil the loss of genetic diversity. Hence, they underestimate the true biological diversity, and

protecting only the visible biodiversity adds additional level of uncertainty for monitoring and management actions of biodiversity and evolutionary processes [112–115]. Therefore, recent taxonomic and phylogenetic studies are mostly based on morphological and molecular data [116–125]. In this study, we considered the phylogenetic concept of the species and discussed also the previously published morphological characteristics of the species. It should be also noted that in this section, we refer to the name of three genera *Aphanius*, *Aphaniops*, and *Paraphanius* for referring their representative species. By applying a COI barcode library for the tooth-carps fishes of the Old World, we conducted an array of molecular species delimitation analyses (i.e., ABGD [100], SP [102], bPTP [104], and bGMYC [105]) to investigate species boundaries and to compared the results to existing morphology-based taxonomy. Across the entire tree, the number of species-level MOTUs (i.e. molecular operational taxonomic units) varied depending on the technique applied, ranging from 35 in SP to 48 in bPTP. The combination of these methods identified 28 (70%) among 40 nominal species. Several MOTUs did not correspond to nominal species, revealing potential cases of synonymy and the presence of species-level cryptic diversity within nominal species. Nevertheless, all the known tooth-carp species were monophyletic except *Aphaniops dispar*, *Aphaniops stiassnyae*, *Aphanius pluristriatus*, and *Aphanius fontinalis*. The majority of methods used split several nominal species including *Aphaniops dispar*, *Aphaniops stoliczkanus*, *Aphanius fasciatus*, and *Aphanius anatoliae* into the independent lineages, which might reflect deep divergences occurring between populations across these species distributions. The species delimitation methods used here revealed a low diversity of the COI barcode region in several species: (i) *Aphanius fontinalis* with *Aphanius sureyanus*; (ii) *Aphaniops stiassnyae* with *Aphaniops dispar*; and (iii) *Aphanius pluristriatus*, *Aphanius kavirensis*, *Aphanius* sp. from Yazd and *Aphanius mesopotamicus* within the *Aphanius sophiae* group. The complexity of the *A*. *sophiae* species group has already been discussed in detail [19]. According to Esmaeili et al. [19], the *A*. *sophiae* subclade includes the most diverse subclade of inland and inland-related species (IIRAS). These authors subdivided the *A*. *sophiae* subclade into three lineages according to their temporal diversification including i) the lineage of *Aphanius isfahanensis* containing only this species, which diverged much earlier (4.8. m.y.a. according to Hrbek et al. [40]) than all other species of the *A*. *sophiae* subclade, ii) *Aphanius farsicus* + *Aphanius arakensis* which probably diverged in the Late Pleistocene (100,000–11,700 y. ago), and iii) the lineage of *A*. *mesopotamicus* comprising a group of very closely related species (*A*. *sophiae*, *A*. *mesopotamicus*, *A*. *pluristriatus*, *A*. *kavirensis*) that may have diversified 11,700 to 4,000 y. ago (Early to Middle Holocene) (see also Gholami et al. [48]). Notably, external characters do not unambiguously distinguish between the species of the *A*. *sophiae* subclade, with the exception of *A*. *isfahanensis*. However, differences in cytochrome *b* sequences and also between the otoliths clearly show that the species of the *A*. *sophiae* subclade are distinct [47–48]. In addition, they inhabit widely separated distribution areas without any hydrological networks or connectivity between, which promotes speciation (Fig 1). These are the reasons that we consider the species of the *A*. *sophiae* subclade as distinct species rather than as populations of a single species.

Moreover, Geiger et al. [86] and Behrens-Chapuis et al. [126] showed that the COI barcode region could not resolve a notable number of fish species. This situation is also usually found in other animal groups [3, 127–129]. The disagreement between distances in the COI barcoding gene sequence and morphologically recognized species might be due to introgressive hybridization, fast evolution, recent speciation events (young group) and genetic drift [86, 126, 130].

Applying molecular species delimitation methods revealed high genetic diversity in *Aphaniops ginaonis*, a species restricted to Genow hot spring in the Hormuzgan basin, southern Iran. This spring is geographically very close to another endemic tooth-carp in this basin,

Aphaniops hormuzensis. Variation in the population of *A*. *ginaonis* has already been noticed and discussed by considering otolith morphology [131]. In the study of Reichenbacher et al. [131], some otoliths showed an atypical morphology i.e., a reduced rostrum length and a lower or higher length/height value. The presence of these otoliths highlighted the possibility of hybridization between *A*. *ginaonis* and *A*. *hormuzensis*. Here, such variability is observed in the COI barcode region of studied *A*. *ginaonis* individuals. Since this spring is currently used by local people for hydrotherapy, possible hybridization cannot be ruled out in this hot spring.

Systematics and biogeography of the *Aphaniops dispar* group have already been discussed by Teimori et al. [15]. The most common species in the family Aphaniidae is *A*. *dispar* and it has long been regarded as a species group rather than a single species. Based on the data provided by Teimori et al. [15] and Freyhof et al. [10], nine species are recognized in the *A*. *dispar* group. The "true" *A*. *dispar* from the Red and the Mediterranean Sea basins, *Aphaniops stoliczkanus* from the coastal areas of the Persian Gulf, the northern Arabian Sea east to Gujarat in India, the Gulf of Oman and some endorheic basins in Iran and Pakistan, *Aphaniops richardsoni* from spring-stream systems in the Dead Sea basin, *Aphaniops sirhani* from the Azraq Oasis in Jordan, *A*. *ginaonis* from a single spring in Hormuzgan basin S-Iran, *Aphaniops furcatus* from a few streams and springs in Hormuzgan basin S- Iran, *A*. *hormuzensis* from Hormuzgan basin S- Iran, *A*. *stiassnyae* from one lake in Ethiopia and *Aphaniops kruppi* from the Wadi Al Batha drainage in northern Oman. Hence, based on the Teimori et al. [15], Freyhof et al. [10], and the data presented here, especially molecular species delimitation methods using the COI barcode region (Fig 2) for *Aphaniops dispar*, the hypothesis of the presence of a single widespread coastal species in the Middle East is rejected and makes it possible that more additional unidentified species occur in the Red Sea basin.

Since biodiversity is in decline due to climate change and anthropogenic land use [132, 133], species are vanishing at a distressing rate while a growing body of evidence underpins the considerable impact of biodiversity loss on the functioning of ecosystems [134–136]. This is also true in the case of the studied fishes, which are the only members of the tooth-carps in the Old World [16], and most of their species are endemic to the landlocked water systems and are under threat owing to natural and human-induced disturbances [14]. These disturbances even forced some species, such as *Aphanius farsicus*, to the edge of extinction [137]. In this context, the efficiency of species identification in some aphaniids was demonstrated in the present study by DNA barcoding and emphasized that COI sequencing could be used to reconstruct the evolutionary relationships within the family Aphaniidae.

Taxonomic remarks on the genera *Aphanius*, *Aphaniops*, and *Paraphanius*

As suggested by Hrbek & Meyer [11], the genus *Aphanius* could be ecologically divided into two main groups: The first group contains those species inhabiting brackish water and euryhaline coastal environments such as the members of *A*. *dispar* group. The second group includes those species inhabiting freshwater systems such as spring-streams, creeks, marshes, and lakes in landlocked basins. Good examples for the latter are those species inhabiting the inland water systems in Anatolia and Iran. The molecular-based study of Hrbek & Meyer [11], highlighted two major phylogenetic clades for the Palearctic killifishes that correspond to the former eastern and western Tethys Sea. Within the eastern clade, two distinct groups have been recognized. One was the freshwater group inhabiting the Arabian Peninsula and the second one was an euryhaline group inhabiting the coastal regions from Pakistan to Somalia. Within the western clade, they recognized a group containing the killifishes of the Iberian Peninsula and the Atlas Mountains, and Turkey and Iran. Based on the tree topology presented here, the *Paraphanius mento* group (*P*. *alexandri*, *P*. *mento*, *P*. *mentoides*, *P*. *orontis*, *P*. *similis*,

and *Paraphanius* sp.) make a distinct clade sister to *Aphaniops dispar* plus the inland and inland-related tooth-carps/*A. fasciatus* groups. Parenti [28], has been summarized several diagnostic features for *Aphanius mento* (now *Paraphanius mento*) that are not found in the members of *Aphanius* and *Aphaniops*, including a cartilaginous interhyal (ossified in *Aphanius* and *Aphaniops*), an embedded urohyal (not embedded in *Aphanius* and *Aphaniops*, see also Teimori et al. [59]), an upturned lower jaw (not upturned in other *Aphanius* and *Aphaniops*), and a distinctive neuromast pattern on the dorsal surface of the head (less prominently developed in *Aphanius* and *Aphaniops* species). Therefore, she proposed *A. mento* as a derived member of the *Aphanius*-like clade that should be designated as "*Aphanius*". This taxonomic separation has later been supported by the observation of the sulcus morphology of the otoliths (Reichenbacher et al. [29], see below).

A study by Reichenbacher et al. [29] used the shape of the otolith sulcus and provided a parameter for the preliminary assignment of the Old World cyprinodontiforms to distinct groups. Based on their study, the Old World tooth-carps can be assigned into two groups: Group I consists of the otoliths with straight sulcus (Fig 4 in Reichenbacher et al. [29]) and includes the Mediterranean species *Aphanius baeticus*, *A. iberus*, *A. fasciatus*, *A. mento* (now *Paraphanius mento*), and the Turkish species; Group II consists of the otoliths with a sulcus distinctly bent terminally (Fig 7 in Reichenbacher et al. [29]). The latter group includes the tooth-carps from the Arabian Peninsula, *A. ginaonis* (now *Aphaniops ginaonis*), and *A. sirhani* (now *Aphaniops sirhani*).

Hrbek & Meyer [11] sorted one species from Group I, i.e., *Aphanius mento* (now *Paraphanius mento*), into the brackish water group consisting of *A. dispar*, *A. ginaonis*, and *A. sirhani*, which represent the Group II in the study of Reichenbacher et al. [29] (see above). As a result, Reichenbacher et al. [29] considered *A. mento* to be an intermediate form between the two groups or clades. In the present study, the phylogenetic analyses revealed that a clade containing *Paraphanius mento* (plus *P. orontis*, *P. mentoides*, *P. alexandri*, *A. similis*, *Paraphanius* sp.), has a basal position in the phylogenetic tree of the Old World tooth-carps, and it is sister to the brackish water *A. dispar* group plus the clade containing inland and inland-related tooth-carps/*A. fasciatus* group. This phylogenetic separation is not completely consistent with the molecular phylogeny provided by Hrbek & Meyer [11], and also partly with the outcomes of Reichenbacher et al. [29].

In addition to the *P. mento* clade, in our DNA barcoding analysis on the Old World tooth-carps, two other large groups were recognized. The first one was the *A. dispar*-like brackish water tooth-carps containing also *A. furcatus* (a brackish water scaleless tooth-carp) and *A. sirhani*. The second group contains all the inland and inland related tooth-carps plus *A. fasciatus* and *A. apodus*. It should be noted that all the inland tooth-carps from Anatolia, Turkey, and Iran fall in the second group, and each of them formed a monophyletic group. This pattern of divergence has proved the hypothesis that all of the Old World tooth-carps originated from an ancestor populated the coastal environments.

Here, we listed several osteological features that show differences between these two large groups (i.e., the members of genus *Aphaniops* vs. those members of the genus *Aphanius*). (i) The number of total vertebrae in *Aphaniops* varies from 25 to 28, verses 26 to 29 in *Aphanius*, (ii) the vertebral columns in *Aphaniops* are often straight versus often slightly curved in *Aphanius*, (iii) the total number of principle caudal-fin rays (PCFR) varies from 9 to 12 (often 10) in *Aphaniops* versus 10–12 (often 11) in *Aphanius*, (iv) the proximal limit of the parhypural in *Aphaniops* is placed very close or has a moderate distance to preural centrum 1, while it has often a clear distance to preural centrum 1 in *Aphanius*, (v) the presence of a constriction on the proximal portion of the neural spine of the preural centrum 2–4 in *Aphaniops*, whereas no obvious constriction presents on the proximal portion of the hemal spine of the preural

centrum in *Aphanius*, (vi) the neural and hemal spines of preural centrum 4 supports at least some caudal fin rays in *Aphaniops*, while they do not reach and support the caudal fin rays in *Aphanius*.

The taxonomic status based on morphology shows the presence of three distinct genera in the family Aphaniidae is confirmed with genetic distances. The average genetic distance among the populations and subspecies is 0.0137% and 0.171%, respectively [138]. Among the genera, this value ranges from 7.70% to 30.50% (mean 23.70%), and within the family, it ranges from 17% to 31% (mean 25%) [138]. In this study, the average K2P genetic distance between a group containing *P. mento* versus a group containing brackish water taxa was 17%, while the average K2P genetic distance between a group containing *P. mento* versus a group containing the inland and inland-related taxa was 19.6% (Table 3). The mean genetic K2P distance within the brackish water group, within the inland and inland-related group, and within the *P. mento* group were 3.7% (S.E. = 0.4%), 13.1% (S.E. = 1%) and 4.8% (S.E. = 0.5%), respectively (Table 4).

Based on the literature review given in the manuscript, morphological comparisons among the distinct groups of the Old World tooth-carps, and species delimitation approached examined in this study, we recognized the genera *Aphanius* Nardo, 1827 and *Aphaniops* Hoedeman, 1951 as valid. The type species for the genus *Aphanius* Nardo, 1827 is *Aphanius nanus* Nardo, 1827 (= *A. fasciatus*) by subsequent designation of Jordan [25]. The type species for the genus *Aphaniops* is *Lebias dispar* Rüppel, 1829 [111].

The genus *Aphanius* contains all the inland and inland-related tooth-carps, which are mainly distributed in the inland waters in Turkey and Iran and also in the inland-related environments around the Mediterranean basin (Fig 2). The genus *Aphaniops* contains all the brackish water in the coastal lines around the Red Sea and the Persian Gulf basins (Fig 2).

Parenti [28] used the *Aphanius mento*-complex to include *mento* (Heckel) and *chantrei* (Gaillard, 1895). Also, Parenti [28] placed the genus name *Aphanius* in parentheses because she noted several deviations from "normal" *Aphanius* species concerning the following characteristics: urohyal embedded in the fold of the branchiostegal membranes and a derived head region pore pattern. As mentioned above, some otolith features have also been reported by Reichenbacher et al. [29] to be unique in *A. mento* and not find in other species of the genus *Aphanius*. Thus, they proposed *A. mento* to be an intermediate form between the two *Aphanius* groups (*Aphanius* and *Aphaniops* in this study).

Parenti [28, 139] places this species in the genus "*Aphanius*", i.e. distinct from true *Aphanius* without defining and naming a new genus. This genus is more closely related to the Anatolian *Kosswigichthys* Sözer, 1942 and the South American *Orestias* Valenciennes in Cuvier and Valenciennes, 1846 than true *Aphanius*.

Comparative material. See S1 Appendix for the list of comparative material examined. The list includes those materials examined by Esmaeili et al. ([50]: *farsicus*), Esmaeili et al. ([19]: *A. darabensis* and *A. kavirensis*); Teimori et al. ([47]: *A. arakensis*), Gholami et al. ([48]: *A. shirini*); Gholami et al. ([20, 80]: *A. farsicus* and *A. sophiae*), Teimori et al. ([51]: *A. furcatus*) and Teimori et al. ([15]: *A. dispar* group).

Nomenclatural acts. The electronic edition of this article conforms to the requirements of the amended International Code of Zoological Nomenclature, and hence the new names contained herein are available under that Code from the electronic edition of this article. This published work and the nomenclatural acts it contains have been registered in ZooBank, the online registration system for the ICZN. The ZooBank LSIDs (Life Science Identifiers) can be resolved and the associated information viewed through any standard web browser by appending the LSID to the prefix "http://zoobank.org/". The LSID for this publication is: urn:lsid:zoobank.org:pub:A8F5EA21-50CE-4FBE-969C-EA6B61FFBABE

Supporting information

S1 Appendix. List of comparative material examined.
(DOCX)

S1 Table. Mean K2P distances between the studied tooth-carps within the family Aphaniidae.
(XLS)

Acknowledgments

We are pleased to thank U. Schliewen and D. Neumann, Department of Ichthyology, Bavarian Natural History Collections, SNSB Bavarian State Collection of Zoology (ZSM) for providing X-ray photography facilities. We thank Z. Gholami helping with DNA extraction. We thank R. Zamanian Nejad, S. Babaee, R. Khaefi, M. Masoudi, and H.R. Mehraban helping with fish collection. We thank B.W. Coad for editing the ms. The research work was approved by the Ethics Committee of Biology Department (SU-9233856).

Author Contributions

Conceptualization: Hamid Reza Esmaeili, Azad Teimori.

Data curation: Hamid Reza Esmaeili, Azad Teimori, Fatah Zarei, Golnaz Sayyadzadeh.

Formal analysis: Hamid Reza Esmaeili, Azad Teimori, Fatah Zarei, Golnaz Sayyadzadeh.

Funding acquisition: Hamid Reza Esmaeili.

Investigation: Hamid Reza Esmaeili, Azad Teimori.

Methodology: Hamid Reza Esmaeili, Azad Teimori, Fatah Zarei, Golnaz Sayyadzadeh.

Software: Hamid Reza Esmaeili, Fatah Zarei.

Supervision: Hamid Reza Esmaeili.

Writing – original draft: Hamid Reza Esmaeili, Fatah Zarei, Golnaz Sayyadzadeh.

Writing – review & editing: Hamid Reza Esmaeili, Azad Teimori, Fatah Zarei, Golnaz Sayyadzadeh.

References

1. Wheeler QD. Taxonomic triage and the poverty of phylogeny. Philosophical Transactions of the Royal Society of London Series B: Biological Sciences. 2004; 359(1444):571–583. https://doi.org/10.1098/rstb.2003.1452 PMID: 15253345
2. de Queiroz K. Species concepts and species delimitation. Systematic Biology. 2007; 56(6):879–886. https://doi.org/10.1080/10635150701701083 PMID: 18027281
3. Hebert PDN, Cywinska A, Ball SL, Dewaard JR. Biological identifications through DNA barcodes. Proceedings of the Royal Society of London Series B: Biological Sciences. 2003; 270(1512):313–321. https://doi.org/10.1098/rspb.2002.2218 PMID: 12614582
4. Ward RD, Zemlak TS, Innes BH, Last PR, Hebert PDN. DNA barcoding Australia's fish species. Philosophical Transactions of the Royal Society B: Biological Sciences. 2005; 360(1462):1847–1857. https://doi.org/10.1098/rstb.2005.1716 PMID: 16214743
5. Ivanova NV, Zemlak TS, Hanner RH, Hebert PDN. Universal primer cocktails for fish DNA barcoding. Molecular Ecology Notes. 2007; 7(4):544–548. https://doi.org/10.1111/j.1471-8286.2007.01748.x
6. Ratnasingham S, Hebert PD. BOLD: The barcode of life data system (www.barcodinglife.org). Molecular Ecology Notes. 2007; 7:355–364. https://doi.org/10.1111/j.1471-8286.2007.01678.x PMID: 18784790

7. Landi M, Dimech M, Arculeo M, Biondo G, Martins R, Carneiro M, et al. DNA barcoding for species assignment: the case of Mediterranean marine fishes. PLoS one. 2014; 9(9):e106135. https://doi.org/10.1371/journal.pone.0106135 PMID: 25222272

8. Gaudant J, Sendrós JR. Découverte de la plus ancienne espèce connue du genre *Aphanius* Nardo (Poissons téléosténns), dans le Miocène inférieur évaporitique de la Catalogne. Batalleria. 1998; 1998 (8):55–60.

9. Freyhof J, Özuluğ M, Saç G. Neotype designation of *Aphanius iconii*, first reviser action to stabilise the usage of *A. fontinalis* and *A. meridionalis* and comments on the family group names of fishes placed in Cyprinodontidae (Teleostei: Cyprinodontiformes). Zootaxa. 2017; 4294(5):573–585. http://dx.doi.org/10.11646/zootaxa.4294.5.6

10. Freyhof J, Weissenbacher A, Geiger M. *Aphanius kruppi*, a new killifish from Oman with comments on the A. dispar species group (Cyprinodontiformes: Aphaniidae). Zootaxa. 2017; 4338(3):557–573. https://doi.org/10.11646/zootaxa.4338.3.10 PMID: 29245719

11. Hrbek T, Meyer A. Closing of the Tethys Sea and the phylogeny of Eurasian killifishes (Cyprinodontiformes: Cyprinodontidae). Journal of Evolutionary Biology. 2003; 16(1):17–36. https://doi.org/10.1046/j.1420-9101.2003.00475.x PMID: 14635877

12. Kosswig C. Zoogeography of the near East. Systematic Biology. 1955; 4(2):49–73. https://doi.org/10.2307/sysbio/4.2.49

13. Sethi RP. Osteology and phylogeny of oviparous cyprinodont fishes (order Cyprinodontiformes). PhD Thesis, University of Florida. 1960.

14. Esmaeili HR, Coad BW, Gholamifard A, Nazari N, Teimory A. Annotated checklist of the freshwater fishes of Iran. Zoosystematica Rossica. 2010; 19(2):361–386.

15. Teimori A, Esmaeili HR, Hamidan N, Reichenbacher B. Systematics and historical biogeography of the *Aphanius dispar* species group (Teleostei: Aphaniidae) and description of a new species from Southern Iran. Journal of Zoological Systematics and Evolutionary Research. 2018; 56(4):579–598. https://doi.org/10.1111/jzs.12228

16. Wildekamp RH. A world of killies: atlas of the oviparous cyprinodontiform fishes of the World, Vol. I. Indiana: American Killifish Association; 1993.

17. Villwock W. Das genus *Aphanius* Nardo, 1827. Deutsche Killifische Gemeinschaft Journal. 1977; 9 (11):165–185.

18. Coad BW. Distribution of *Aphanius* species in Iran. The Journal of the American Killifish Association. 2000; 33:183–191.

19. Esmaeili HR, Teimori A, Gholami Z, Reichenbacher B. Two new species of the tooth-carp *Aphanius* (Teleostei: Cyprinodontidae) and the evolutionary history of the Iranian inland and inland-related Aphanius species. Zootaxa. 2014; 3786(3):246–268. https://doi.org/10.11646/zootaxa.3786.3.2 PMID: 24869537

20. Gholami Z, Esmaeili HR, Erpenbeck D, Reichenbacher B. Genetic connectivity and phenotypic plasticity in the cyprinodont *Aphanius* farsicus from the Maharlu Basin, south-western Iran. Journal of Fish Biology. 2015; 86(3):882–906. https://doi.org/10.1111/jfb.12599 PMID: 25644025

21. Teimori A, Jawad LAJ, Al-Kharusi LH, Al-Mamry JM, Reichenbacher B. Late Pleistocene to Holocene diversification and historical zoogeography of the Arabian killifish (*Aphanius dispar*) inferred from otolith morphology. Scientia Marina. 2012; 76(4):637–645. https://doi.org/10.3989/scimar.03635.26C

22. Teimori A, Schulz-Mirbach T, Esmaeili HR, Reichenbacher B. Geographical differentiation of *Aphanius dispar* (Teleostei: Cyprinodontidae) from southern Iran. Journal of Zoological Systematics and Evolutionary Research. 2012; 50(4):289–304. https://doi.org/10.1111/j.1439-0469.2012.00667.x

23. WİIdekamp RH, Küçük F, Ünlüsayin M, Van Neer W. Species and subspecies of the genus *Aphanius* Nardo 1897 (Pisces: Cyprinodontidae) in Turkey. Turkish Journal of Zoology. 1999; 23(1):23–44.

24. Kottelat M, Wheeler AC. *Aphanius* Nardo, 1827 (Osteichthyes, Cyprinodontiformes): proposed placement on the Official List. Bulletin of Zoological Nomenclature. 2001; 58:110–115.

25. Jordan DS. The genera of fishes: from linnaeus to Cuvier, 1758–1833, seventy-five years, with the accepted type of each, a contribution to the stability of scientific nomenclature. Stanford University: Leland Stanford Jr. University Publications; 1917.

26. Lazara KJ. History of the genera *Lebia* Oken 1817 and *Lebias* Goldfuss 1820 (Teleostei: Cyprinodontiformes: Cyprinodontidae) with designation of a type species for *Lebias*. Copeia. 1995; 1995(2):501–503. https://doi.org/10.2307/1446923

27. Parenti LR. A phylogenetic and biogeographic analysis of cyprinodontiform fishes (Teleostei, Atherinomorpha). Bulletin of the American Museum of Natural History. 1981; 168:335–557.

28. Reichenbacher B, Sienknecht U, Küchenhoff H, Fenske N. Combined otolith morphology and morphometry for assessing taxonomy and diversity in fossil and extant killifish (*Aphanius*,†*Prolebias*). Journal of Morphology. 2007; 268(10):898–915. https://doi.org/10.1002/jmor.10561 PMID: 17674357

29. Lazara KJ. The killifishes, an annotated checklist, synonymy, and bibliography of recent oviparous Cyprinodontiform fishes. The killifish master index. Indiana: American Killifish Association; 2001.

30. Hoedeman JJ. Rediagnosis of the old world cyprinodont genus *Aphanius*. Beaufortia. 1951; 1(1):1–6.

31. Tortonese E. [Various family accounts.]. In: Hureau J-C, Monod T, editors. Check-list of the fishes of the north-eastern Atlantic and of the Mediterranean. Paris: UNESCO; 1973.

32. Wildekamp RH, Romand R, Scheel JJ. Cyprinodontidae. Check-list of the freshwater fishes of Africa (CLOFFA). 2. Paris: ORSTOM; 1986. pp. 165–276.

33. Coad BW. *Aphanius vladykovi*, a new species of tooth-carp from the Zagros Mountains of Iran (Osteichthyes: Cyprinodontidae). Environmental Biology of Fishes. 1988; 23(1):115–126. https://doi.org/10.1007/BF00000741

34. Krupp F, Schneider W. The fishes of the Jordan River drainage basin and Azraq Oasis. Fauna of Saudi Arabia. 1989; 10:347–416.

35. Poll M, Gosse J-P. Genera des poissons d'eau douce de l'Afrique. Mémoire de la Classe des Sciences. 1995; 9:1–324.

36. Seegers L. Aqualog: killifishes of the World: Old World killis II. Germany: Aqualog, Verlag A.C.S., Mörfelden-Walldorf; 1997.

37. Doadrio I, Carmona JA, Fernandez-Delgado C. Morphometric study of the Iberian *Aphanius* (Actinopterygii, Cyprinodontiformes), with description of a new species. Folia Zoologica. 2002; 51(1):67–80.

38. Huber JH. A review of family-group names for oviparous Cyprinodontiformes (Pisces: Teleostei). UK: British Killifish Association, Separatum; 2005.

39. Jl Blanco, Hrbek T, Doadrio I. A new species of the genus *Aphanius* (Nardo, 1832)(Actinopterygii, Cyprinodontidae) from Algeria. Zootaxa. 2006; 1158(1):39–53. https://doi.org/10.11646/zootaxa.1158.1.2

40. Hrbek T, Keivany Y, Coad BW. New species of *Aphanius* (Teleostei, Cyprinodontidae) from Isfahan Province of Iran and a reanalysis of other Iranian species. Copeia. 2006; 2006(2):244–255. https://doi.org/10.1643/0045-8511(2006)6[244:NSOATC]2.0.CO;2

41. Kottelat M, Barbieri R, Stoumboudi MT. *Aphanius almitiensis*, a new species of toothcarp from Greece (Teleostei: Cyprinodontidae). Revue Suisse de Zoologie. 2007; 114(1):13–31. https://doi.org/10.5962/bhl.part.80385

42. Kottelat M, Freyhof Jr. Handbook of European freshwater fishes. Cornol, Switzerland: Publications Kottelat; 2007.

43. Hertwig ST. Phylogeny of the Cyprinodontiformes (Teleostei, Atherinomorpha): the contribution of cranial soft tissue characters. Zoologica Scripta. 2008; 37(2):141–174. https://doi.org/10.1111/j.1463-6409.2007.00314.x

44. Coad B. A new species of tooth-carp, *Aphanius mesopotamicus*, from Iran and Iraq (Actinopterygii, Cyprinodontidae). Zookeys. 2009; 31:149. https://doi.org/10.3897/zookeys.31.131

45. Teimori A, Esmaeili HR, Reichenbacher B. *Aphanius farsicus*, a replacement name for A. persicus (Jenkins, 1910)(Teleostei, Cyprinodontidae). Zootaxa. 2011; 3096(1):53–58. https://doi.org/10.11646/zootaxa.3096.1.5

46. Esmaeili HR, Teimori A, Gholami Z, Zarei N, Reichenbacher B. Re-validation and re-description of an endemic and threatened species, *Aphanius pluristriatus* (Jenkins, 1910)(Teleostei, Cyprinodontidae), from southern Iran. Zootaxa. 2012; 3208(1):58–67. https://doi.org/10.11646/zootaxa.3208.1.4

47. Teimori A, Esmaeili HR, Gholami Z, Zarei N, Reichenbacher B. *Aphanius arakensis*, a new species of tooth-carp (Actinopterygii, Cyprinodontidae) from the endorheic Namak Lake basin in Iran. Zookeys. 2012; 215:55–76. https://doi.org/10.3897/zookeys.215.1731 PMID: 22936871

48. Gholami Z, Esmaeili HR, Erpenbeck D, Reichenbacher B. Phylogenetic analysis of *Aphanius* from the endorheic Kor River Basin in the Zagros Mountains, South-western Iran (Teleostei: Cyprinodontiformes: Cyprinodontidae). Journal of Zoological Systematics and Evolutionary Research. 2014; 52(2):130–141. https://doi.org/10.1111/jzs.12052

49. Keivany Y, Esmaeili HR. Threatened fishes of the world: *Aphanius pluristriatus* (Jenkins 1910)(Cyprinodontidae). Aqua: International Journal of Ichthyology. 2014; 20(2):67–73.

50. Esmaeili HR, Teimori A, Sayyadzadeh G, Masoudi M, Reichenbacher B. Phylogenetic relationships of the tooth-carp *Aphanius* (Teleostei: Cyprinodontidae) in the river systems of southern and south-western Iran based on mtDNA sequences. Zoology in the Middle East. 2014; 60(1):29–38. https://doi.org/10.1080/09397140.2014.892329

51. Teimori A, Esmaeili HR, Erpenbeck D, Reichenbacher B. A new and unique species of the genus *Aphanius* Nardo, 1827 (Teleostei: Cyprinodontidae) from Southern Iran: A case of regressive evolution. Zoologischer Anzeiger-A Journal of Comparative Zoology. 2014; 253(4):327–337. https://doi.org/10.1016/j.jcz.2013.12.001

52. Pfleiderer SJ, Geiger MF, Herder F. *Aphanius marassantensis*, a new toothcarp from the Kızılırmak drainage in northern Anatolia (Cyprinodontiformes: Cyprinodontidae). Zootaxa. 2014; 3887(5):569–582. https://doi.org/10.11646/Zootaxa.3887.5.4 PMID: 25543950

53. Huber JH. Comments on the description of *Aphanius marassantensis* by Pfleiderer, Geiger & Herder, 2014, with proposed synonymization. Killi-Data. 2015; 2015:31–35.

54. Jouladeh-Roudbar A, Vatandoust S, Eagderi S, Jafari-Kenari S, Mousavi-Sabet H. Freshwater fishes of Iran; an updated checklist. AACL Bioflux. 2015; 8(6):855–909.

55. Esmaeili HR, Mehraban H, Abbasi K, Keivany Y, Brian WC. Review and updated checklist of freshwater fishes of Iran: Taxonomy, distribution and conservation status. Iranian Journal of Ichthyology. 2017; 4(Suppl. 1):1–114.

56. Çiçek E, Fricke R, Sungur S, Eagderi S. Endemic freshwater fishes of Turkey. FishTaxa. 2018; 3 (4):1–39.

57. Esmaeili HR, Sayyadzadeh G, Eagderi S, Abbasi K. Checklist of freshwater fishes of Iran. FishTaxa. 2018; 3(3):1–95.

58. Golani D, Fricke R. Checklist of the Red Sea fishes with delineation of the Gulf of Suez, Gulf of Aqaba, endemism and Lessepsian migrants. Zootaxa. 2018; 4509(1):1–215. https://doi.org/10.11646/zootaxa.4509.1.1 PMID: 30485948

59. Teimori A, Motamedi M, Iranmanesh A. Comparative morphology of urohyal bone in brackish water species of the genus *Aphanius* Nardo, 1827 in the Persian Gulf and Southeastern Mediterranean Sea basins (Teleostei: Aphaniidae). Mediterranean Marine Science. 2018; 19(2):356–365. http://dx.doi.org/10.12681/mms.15929

60. Yoğurtçuoğlu B, Freyhof J. *Aphanius irregularis*, a new killifish from south-western Anatolia (Cyprinodontiformes: Aphaniidae). Zootaxa. 2018; 4410(2):319–330. https://doi.org/10.11646/zootaxa.4410.2.4 PMID: 29690146

61. Teimori A, Motamedi M. The first complete mitochondrial genome sequence in the genus *Aphanius* (Teleostei). Journal of Ichthyology. 2019; 59(5):754–765. https://doi.org/10.1134/S0032945219050151

62. Motamedi M, Teimori A, Iranmanesh A. Ontogenetic pattern, morphological sexual and side dimorphism in the saccular otolith of a scaleless killifish *Aphanius furcatus* (Teleostei: Aphaniidae). Acta Zoologica. 2019; 00:1–13. https://doi.org/10.1111/azo.12313

63. Costa WJEM. Phylogeny and classification of the Cyprinodontidae revisited (Teleostei: Cyprinodontiformes): are Andean and Anatolian killifishes sister taxa. Journal of Comparative Biology. 1997; 2 (1):1–17.

64. Nelson JS, Grande TC, Wilson MVH. Fishes of the World. New Jersey: John Wiley & Sons, Inc.; 2016.

65. Fricke R, Eschmeyer WN, van der Laan R. Eschmeyer's catalog of fishes: genera, species, references. http://researcharchive.calacademy.org/research/ichthyology/catalog/fishcatmain.asp. Electronic version accessed 20 August 2019. 2019.

66. Coad BW, Abdoli A. Systematics of an isolated population of tooth-carp from northern Iran (Actinopterygii: Cyprinodontidae). Zoology in the Middle East. 2000; 21(1):87–102. https://doi.org/10.1080/09397140.2000.10637836

67. Chiozzi G, Stiassny MLJ, Alter SE, De Marchi G, Mebrahtu Y, Tessema M, et al. Fishes in the desert: mitochondrial variation and phylogeography of *Danakilia* (Actinopterygii: Cichlidae) and *Aphanius* (Actinopterygii: Cyprinodontidae) in the Danakil Depression of northeastern Africa. Mitochondrial DNA Part A. 2018; 29(7):1025–1040. https://doi.org/10.1080/24701394.2017.1404043 PMID: 29166850

68. Ferrito V, Mannino MC, Pappalardo AM, Tigano C. Morphological variation among populations of *Aphanius fasciatus* Nardo, 1827 (Teleostei, Cyprinodontidae) from the Mediterranean. Journal of Fish Biology. 2007; 70(1):1–20. https://doi.org/10.1111/j.1095-8649.2006.01192.x

69. Ferrito V, Pappalardo AM, Canapa A, Barucca M, Doadrio I, Olmo E, et al. Mitochondrial phylogeography of the killifish *Aphanius fasciatus* (Teleostei, Cyprinodontidae) reveals highly divergent Mediterranean populations. Marine Biology. 2013; 160(12):3193–3208. http://dx.doi.org/10.1007/s00227-013-2307-4

70. Tigano C, Ferrito V, Nicosia R. Morphological analysis of the pharyngeal jaws in two populations of *Lebias fasciata* Valenciennes, 1821 (Teleostei: Cyprinodontidae). Journal of Morphology. 1999; 241

(2):107–114. https://doi.org/10.1002/(SICI)1097-4687(199908)241:2<107::AID-JMOR1>3.0.CO;2-0 PMID: 10420157

71. Tigano C, Canapa A, Ferrito V, Barucca M, Arcidiacono I, Deidun A, et al. A study of osteological and molecular differences in populations of *Aphanius fasciatus* Nardo 1827, from the central Mediterranean (Teleostei, Cyprinodontidae). Marine Biology. 2006; 149(6):1539–1550. https://doi.org/10.1007/s00227-006-0300-x

72. Villwock W. *Aphanius* (Nardo, 1827) and *Cyprinodon* (Lac, 1803)(Pisces: Cyprinodontidae), an attempt for a genetic interpretation of speciation. Journal of Zoological Systematics and Evolutionary Research. 1983; 20(3):187–197. https://doi.org/10.1111/j.1439-0469.1983.tb00264.x

73. Esmaeili HR, Zarei F, Vahed NS, Masoudi M. Scale morphology and phylogenetic character mapping of scale-surface microstructures in sixteen *Aphanius* species (Teleostei: Aphaniidae). Micron. 2019; 119:39–53. https://doi.org/10.1016/j.micron.2019.01.002 PMID: 30660090

74. Sanjarani Vahed N, Esmaeili HR, Masoudi M, Reichenbacher B. Early otolith development in the critically endangered tooth-carp, *Aphanius farsicus* (Teleostei: Cyprinodontidae). Environmental Biology of Fishes. 2018; 101(9):1309–1317. https://doi.org/10.1007/s10641-018-0778-3

75. Masoudi M, Esmaeili HR, Ebrahimi M. Embryology and early ontogeny of an endemic tooth-carp fish, *Aphanius sophiae* (Heckel, 1847). Journal of Applied Ichthyology. 2018; 34(3):622–632. https://doi.org/10.1111/jai.13630

76. Sanjarani Vahed N, Esmaeili HR, Masoudi M, Ebrahimi M. Embryonic and early development of the Zagros tooth-carp, *Aphanius vladykovi* (Actinopterygii: Cyprinodontidae). Journal of Morphology. 2018; 279(6):747–756. https://doi.org/10.1002/jmor.20807 PMID: 29464805

77. Sanjarani Vahed N, Esmaeili HR, Masoudi M, Ebrahimi M. Towards the conservation of a critically endangered species, *Aphanius farsicus*: embryogenesis and development. Environmental Biology of Fishes. 2018; 101(1):193–202. https://doi.org/10.1007/s10641-017-0691-1

78. Sanjarani Vahed N, Esmaeili HR, Masoudi M, Reichenbacher B. Ontogenetic otolith development in an endemic tooth-carp, *Aphanius vladykovi* (Teleostei: Aphaniidae). Journal of Ichthyology. 2019; 59 (3):336–343. https://doi.org/10.1134/S0032945219030172

79. Masoudi M, Esmaeili HR, Ebrahimi M, Teimori A, Seifali M. Reproductive biology of two sympatric species of tooth-carps: *Aphanius hormuzensis* and *Aphanius furcatus*, from south of Iran (Teleostei: Aphaniidae). International Journal of Aquatic Biology. 2018; 6(4):189–197.

80. Gholami Z, Esmaeili HR, Reichenbacher B. New data on the zoogeography of *Aphanius sophiae* (Teleostei: Cyprinodontidae) in the Central Zagros (Southwest Iran). Limnologica. 2015; 51:70–82. https://doi.org/10.1016/j.limno.2014.12.002

81. Gholami Z, Teimori A, Esmaeili HR, Schulz-Mirbach T, Reichenbacher B. Scale surface microstructure and scale size in the tooth-carp genus *Aphanius* (Teleostei, Cyprinodontidae) from endorheic basins in Southwest Iran. Zootaxa. 2013; 3619(4):467–490. https://doi.org/10.11646/zootaxa.3619.4.5 PMID: 26131487

82. Masoudi M, Esmaeili HR, Teimori A, Gholami Z, Gholamhosseini A, Sayyadzadeh G, et al. Sympatry and possible hybridization among species of the killifish genus *Aphanius* Nardo, 1827 (Teleostei: Cyprinodontidae) in Southwestern Iran. Limnologica. 2016; 59:10–20. https://doi.org/10.1016/j.limno.2016.02.008

83. Coad BW. A re-description of *Aphanius ginaonis* (Holly, 1929) from southern Iran (Osteichthyes: Cyprinodontiformes). Journal of Natural History. 1980; 14(1):33–40. https://doi.org/10.1080/00222938000770031

84. Vargas S, Schuster A, Sacher K, Büttner G, Schätzle S, Läuchli B, et al. Barcoding sponges: an overview based on comprehensive sampling. PloS one. 2012; 7(7):e39345. https://doi.org/10.1371/journal.pone.0039345 PMID: 22802937

85. Geiger MF. FREDIE—Freshwater Diversity Identification for Europe. Unpublished data on GenBank. 2018.

86. Geiger MF, Herder F, Monaghan MT, Almada V, Barbieri R, Bariche M, et al. Spatial heterogeneity in the Mediterranean Biodiversity Hotspot affects barcoding accuracy of its freshwater fishes. Molecular Ecology Resources. 2014; 14(6):1210–1221. https://doi.org/10.1111/1755-0998.12257 PMID: 24690331

87. Rabaoui L, Lin Y-J, Qurban MA, Shen K-N. Fishes of the Arabian Gulf. Unpublished data on GenBank. 2016.

88. Webb SA, Graves JA, Macias-Garcia C, Magurran AE, Foighil DO, Ritchie MG. Molecular phylogeny of the livebearing Goodeidae (Cyprinodontiformes). Molecular Phylogenetics and Evolution. 2004; 30 (3):527–544. https://doi.org/10.1016/S1055-7903(03)00257-4 PMID: 15012937

89. Hall TA. BioEdit: a user-friendly biological sequence alignment editor and analysis program for Windows 95/98/NT. Nucleic Acids Symposium Series. 1999; 41(41):95–98.

90. Kumar S, Stecher G, Tamura K. MEGA7: molecular evolutionary genetics analysis version 7.0 for bigger datasets. Molecular Biology and Evolution. 2016; 33(7):1870–1874. https://doi.org/10.1093/molbev/msw054 PMID: 27004904

91. Xia X. DAMBE7: New and improved tools for data analysis in molecular biology and evolution. Molecular Biology and Evolution. 2018; 35:1550–1552. https://doi.org/10.1093/molbev/msy073 PMID: 29669107

92. Ronquist F, Teslenko M, Van Der Mark P, Ayres DL, Darling A, Höhna S, et al. MrBayes 3.2: efficient Bayesian phylogenetic inference and model choice across a large model space. Systematic Biology. 2012; 61(3):539–542. https://doi.org/10.1093/sysbio/sys029 PMID: 22357727

93. Drummond AJ, Ho SY, Phillips MJ, Rambaut A. Relaxed phylogenetics and dating with confidence. PLoS Biology. 2006; 4(5):e88. https://doi.org/10.1371/journal.pbio.0040088 PMID: 16683862

94. Drummond AJ, Suchard MA, Xie D, Rambaut A. Bayesian phylogenetics with BEAUti and the BEAST 1.7. Molecular Biology and Evolution. 2012; 29(8):1969–1973. https://doi.org/10.1093/molbev/mss075 PMID: 22367748

95. Rambaut A, Suchard MA, Xie D, Drummond AJ. Tracer v1.6. Available at: http://beast.bio.ed.ac.uk/Tracer. 2014.

96. Gaudant J. Révision de *Prolebias stenoura* Sauvage, 1874 du Stampien (= Rupélien) de Limagne (centre de la France), espèce type du genre *Prolebias* (poisson téléostéen, Cyprinodontiformes). Geodiversitas. 2012; 34(2):409–423. https://doi.org/10.5252/g2012n2a9

97. Rambaut A, Drummond AJ. TreeAnnotator v1. 7.0. Available at: http://beast.bio.ed.ac.uk/TreeAnnotator. 2013.

98. Stamatakis A. RAxML-VI-HPC: maximum likelihood-based phylogenetic analyses with thousands of taxa and mixed models. Bioinformatics. 2006; 22:2688–2690. https://doi.org/10.1093/bioinformatics/btl446 PMID: 16928733

99. Puillandre N, Lambert A, Brouillet S, Achaz G. ABGD, Automatic Barcode Gap Discovery for primary species delimitation. Molecular Ecology. 2012; 21(8):1864–1877. https://doi.org/10.1111/j.1365-294X.2011.05239.x PMID: 21883587

100. Kimura M. A simple method for estimating evolutionary rates of base substitutions through comparative studies of nucleotide sequences. Journal of Molecular Evolution. 1980; 16(2):111–120. https://doi.org/10.1007/bf01731581 PMID: 7463489

101. Hart MW, Sunday J. Things fall apart: biological species form unconnected parsimony networks. Biology Letters. 2007; 3(5):509–512. https://doi.org/10.1098/rsbl.2007.0307 PMID: 17650475

102. Clement M, Posada D, Crandall KA. TCS: a computer program to estimate gene genealogies. Molecular Ecology. 2000; 9(10):1657–1659. https://doi.org/10.1046/j.1365-294x.2000.01020.x PMID: 11050560

103. Zhang J, Kapli P, Pavlidis P, Stamatakis A. A general species delimitation method with applications to phylogenetic placements. Bioinformatics. 2013; 29(22):2869–2876. https://doi.org/10.1093/bioinformatics/btt499 PMID: 23990417

104. Reid NM, Carstens BC. Phylogenetic estimation error can decrease the accuracy of species delimitation: a Bayesian implementation of the general mixed Yule-coalescent model. BMC Evolutionary Biology. 2012; 12(1):196. https://doi.org/10.1186/1471-2148-12-196 PMID: 23031350

105. Yule G. A mathematical theory of evolution, based on the conclusions of Dr. JC Willis, FRS. Philosophical Transactions of the Royal Society of London Series B. 1925; 213:21–87.

106. Kingman JFC. On the genealogy of large populations. Journal of Applied Probability. 1982; 19(A):27–43. https://doi.org/10.2307/3213548

107. Team RC. R: A language and environment for statistical computing. http://R-Forge.R-project.org. R Foundation for Statistical Computing, Vienna, Austria. 2013.

108. Akşiray F. Türkiye Cyprinodontidleri hakkında I. Türkische Cyprinodontiden I. Revue de la Faculté des Sciences de l'Université d'Istanbul. 1948; 13:97–142.

109. Villwock VW. Genetische Untersuchungen an altweltlichen Zahnkarpfen der Tribus *Aphaniini* (Pisces, Cyprinodontidae) nach Gesichtspunkten der Neuen Systematik. Journal of Zoological Systematics and Evolutionary Research. 1964; 2(4):267–382. https://doi.org/10.1111/j.1439-0469.1964.tb00722.x

110. Villwock W. Weitere genetische Untersuchungen zur Frage der Verwandtschaftsbeziehungen anatolischer Zahnkarpfen. Mitteilungen aus dem Hamburgischen Zoologischen Museum und Institut. 1958; 56:81–153.

111. Briggs JC. Coral reefs: conserving the evolutionary sources. Biological Conservation. 2005; 126 (3):297–305. https://doi.org/10.1016/j.biocon.2005.06.018

112. Moritz C. Strategies to protect biological diversity and the evolutionary processes that sustain it. Systematic Biology. 2002; 51(2):238–254. https://doi.org/10.1080/10635150252899752 PMID: 12028731

113. Moritz C. Conservation units and translocations: strategies for conserving evolutionary processes. Hereditas. 1999; 130(3):217–228. https://doi.org/10.1111/j.1601-5223.1999.00217.x

114. Adams M, Raadik TA, Burridge CP, Georges A. Global biodiversity assessment and hyper-cryptic species complexes: more than one species of elephant in the room?. Systematic Biology. 2014; 63 (4):518–533. https://doi.org/10.1093/sysbio/syu017 PMID: 24627185

115. Bálint M, Domisch S, Engelhardt CHM, Haase P, Lehrian S, Sauer J, et al. Cryptic biodiversity loss linked to global climate change. Nature Climate Change. 2011; 1(6):313–318. https://doi.org/10.1038/nclimate1191

116. Dincă V, Montagud S, Talavera G, Hernández-Roldán J, Munguira ML, García-Barros E, et al. DNA barcode reference library for Iberian butterflies enables a continental-scale preview of potential cryptic diversity. Scientific Reports. 2015; 5:12395. https://doi.org/10.1038/srep12395 PMID: 26205828

117. Féral J-P. How useful are the genetic markers in attempts to understand and manage marine biodiversity?. Journal of Experimental Marine Biology and Ecology. 2002; 268(2):121–145. https://doi.org/10.1016/S0022-0981(01)00382-3

118. Geist J. Integrative freshwater ecology and biodiversity conservation. Ecological Indicators. 2011; 11 (6):1507–1516. https://doi.org/10.1016/j.ecolind.2011.04.002

119. Karp A, Ingram DS, Isaac PG. Molecular tools for screening biodiversity: plants and animals. Springer Science & Business Media; 2012.

120. Mutanen M, Kaila L, Tabell J. Wide-ranging barcoding aids discovery of one-third increase of species richness in presumably well-investigated moths. Scientific Reports. 2013; 3:2901. https://doi.org/10.1038/srep02901 PMID: 24104541

121. Pauls SU, Nowak C, Bálint M, Pfenninger M. The impact of global climate change on genetic diversity within populations and species. Molecular Ecology. 2013; 22(4):925–946. https://doi.org/10.1111/mec.12152 PMID: 23279006

122. Vieites DR, Wollenberg KC, Andreone F, Köhler J, Glaw F, Vences M. Vast underestimation of Madagascar's biodiversity evidenced by an integrative amphibian inventory. Proceedings of the National Academy of Sciences. 2009; 106(20):8267–8272. https://doi.org/10.1073/pnas.0810821106 PMID: 19416818

123. Yannic G, Pellissier L, Ortego J, Lecomte N, Couturier S, Cuyler C, et al. Genetic diversity in caribou linked to past and future climate change. Nature Climate Change. 2014; 4(2):132. https://doi.org/10.1038/nclimate2074

124. Katouzian A-R, Sari A, Macher JN, Weiss M, Saboori A, Leese F, et al. Drastic underestimation of amphipod biodiversity in the endangered Irano-Anatolian and Caucasus biodiversity hotspots. Scientific Reports. 2016; 6:22507. https://doi.org/10.1038/srep22507 PMID: 26928527

125. Behrens-Chapuis S, Herder F, Esmaeili HR, Freyhof J, Hamidan NA, Özuluğ M, et al. Adding nuclear rhodopsin data where mitochondrial COI indicates discrepancies–can this marker help to explain conflicts in cyprinids?. DNA Barcodes. 2015; 3(1):187–199. https://doi.org/10.1515/dna-2015-0020

126. Vences M, Thomas M, Bonett RM, Vieites DR. Deciphering amphibian diversity through DNA barcoding: chances and challenges. Philosophical Transactions of the Royal Society B: Biological Sciences. 2005; 360(1462):1859–1868. https://doi.org/10.1098/rstb.2005.1717 PMID: 16221604

127. Waugh J. DNA barcoding in animal species: progress, potential and pitfalls. BioEssays. 2007; 29 (2):188–197. https://doi.org/10.1002/bies.20529 PMID: 17226815

128. Laurito M, de Oliveira TMP, Almiron WR, Sallum MAM. COI barcode versus morphological identification of *Culex* (*Culex*)(Diptera: Culicidae) species: a case study using samples from Argentina and Brazil. Memórias do Instituto Oswaldo Cruz. 2013; 108:110–122. https://doi.org/10.1590/0074-0276130457 PMID: 24473810

129. Huemer P, Hebert PDN, Mutanen M, Wieser C, Wiesmair B, Hausmann A, et al. Large geographic distance versus small DNA barcode divergence: Insights from a comparison of European to South Siberian Lepidoptera. PloS one. 2018; 13(11):e0206668. https://doi.org/10.1371/journal.pone.0206668 PMID: 30388147

130. Thomas CD, Cameron A, Green RE, Bakkenes M, Beaumont LJ, Collingham YC, et al. Extinction risk from climate change. Nature. 2004; 427(6970):145–148. https://doi.org/10.1038/nature02121 PMID: 14712274

131. Brooks TM, Mittermeier RA, da Fonseca GAB, Gerlach J, Hoffmann M, Lamoreux JF, et al. Global biodiversity conservation priorities. Science. 2006; 313(5783):58–61. https://doi.org/10.1126/science.1127609 PMID: 16825561

132. Wardle DA, Bardgett RD, Callaway RM, Van der Putten WH. Terrestrial ecosystem responses to species gains and losses. Science. 2011; 332(6035):1273–1277. https://doi.org/10.1126/science.1197479 PMID: 21659595

133. Cardinale BJ, Duffy JE, Gonzalez A, Hooper DU, Perrings C, Venail P, et al. Biodiversity loss and its impact on humanity. Nature. 2012; 486(7401):59–67. https://doi.org/10.1038/nature11148 PMID: 22678280

134. Hooper DU, Adair EC, Cardinale BJ, Byrnes JEK, Hungate BA, Matulich KL, et al. A global synthesis reveals biodiversity loss as a major driver of ecosystem change. Nature. 2012; 486(7401):105–108. https://doi.org/10.1038/nature11118 PMID: 22678289

135. Esmaeili HR, Masoudi M, Ebrahimi M, Amir E. Review of *Aphanius farsicus*: a critically endangered species (Teleostei: Cyprinodontidae) in Iran. Iranian Journal of Ichthyology. 2016; 3(1):1–18.

136. Parenti LR. A taxonomic revision of the Andean killifish genus *Orestias* (Cyprinodontiformes, Cyprinodontidae). Bulletin of the American Museum of Natural History. 1984; 178:107–2014.

137. Nei M. Genetic distance between populations. The American Naturalist. 1972; 106(949):283–292.

138. Reichenbacher B, Kamrani E, Esmaeili HR, Teimori A. The endangered cyprinodont *Aphanius ginaonis* (Holly, 1929) from southern Iran is a valid species: evidence from otolith morphology. Environmental Biology of Fishes. 2009; 86(4):507–521. https://doi.org/10.1007/s10641-009-9549-5

139. Darriba D, Taboada GL, Doallo R, Posada D. jModelTest 2: more models, new heuristics and parallel computing. Nature Methods. 2012; 9(8):772. https://doi.org/10.1038/nmeth.2109 PMID: 22847109
