# Supplementary material for: Cryptic biodiversity of freshwater fish species in Bangladesh
Source: PLoS One. 2025 Apr 28;20(4):e0318982. doi: 10.1371/journal.pone.0318982 (PMC12036941; doi:10.1371/journal.pone.0318982)
Supplement: Attachment — Submitted filename: PONE-D-24-38143 (1).pdf [file pone.0318982.s007.pdf]

PLOS ONE

# Cryptic biodiversity of freshwater fish species in Bangladesh

--Manuscript Draft--

| | |
|---|---|
| **Manuscript Number:** | PONE-D-24-38143 |
| **Article Type:** | Research Article |
| **Full Title:** | Cryptic biodiversity of freshwater fish species in Bangladesh |
| **Short Title:** | Cryptic biodiversity of freshwater fish |
| **Corresponding Author:** | Mahmudul Hasan<br>Bangamata Sheikh Fojilatunnesa Mujib Science & Technology University<br>Jamalpur, BANGLADESH |
| **Keywords:** | Cryptic species, freshwater fishes, conservation, Bangladesh |
| **Abstract:** | Unrecognized cryptic species impede conservation planning and biodiversity assessments. DNA barcoding has tremendously expanded the number of novel species and cryptic species in biological science. Despite few sporadic studies, the exact number of freshwater species found in Bangladesh is not known. To assess this biodiversity, we sequenced the COI gene of 124freshwater specimens which were gathered from various locations around Bangladesh. Seven cryptic/candidate species hidden among the currently studied specimens were identified based on the findings of phylogenetic and species delimitation analyses that had included the data that was available for related species. Limited morphological examination was also applied to ensure the certainty of cryptic/candidate species. The appearance of new species, some of them possibly endemic has been hypothesized. This raises concerns regarding the true diversity and evolutionary history of freshwater species in Bangladesh, which are significantly underrepresented in the current systematic frameworks that do not account for DNA data. Our current study provides baseline data might aid local ichthyologists in their quest to identify additional new species by combining several variables (morphology and ecology). Further research is warranted to protect the priceless freshwater species in Bangladesh. |
| **Order of Authors:** | Mahmudul Hasan |
| | Chiaki Kambayashi, PhD |
| | Zahid Hasan Anik |
| | Md. Saiful Islam, MS |
| **Opposed Reviewers:** | |
| **Additional Information:** | |
| **Question** | **Response** |
| **Financial Disclosure**<br><br>Enter a financial disclosure statement that describes the sources of funding for the work included in this submission. Review the submission guidelines for detailed requirements. View published research articles from *PLOS ONE* for specific examples.<br><br>This statement is required for submission and **will appear in the published article** if the submission is accepted. Please make sure it is accurate. | The author(s) received no specific funding for this work. |

*Powered by Editorial Manager® and ProduXion Manager® from Aries Systems Corporation*

| | |
|---|---|
| **Funded studies**<br>Enter a statement with the following details:<br>• Initials of the authors who received each award<br>• Grant numbers awarded to each author<br>• The full name of each funder<br>• URL of each funder website<br>• Did the sponsors or funders play any role in the study design, data collection and analysis, decision to publish, or preparation of the manuscript?<br><br>Did you receive funding for this work? | |
| **Competing Interests**<br><br>Use the instructions below to enter a competing interest statement for this submission. On behalf of all authors, disclose any competing interests that could be perceived to bias this work—acknowledging all financial support and any other relevant financial or non-financial competing interests.<br><br>This statement is required for submission and **will appear in the published article** if the submission is accepted. Please make sure it is accurate and that any funding sources listed in your Funding Information later in the submission form are also declared in your Financial Disclosure statement.<br><br>View published research articles from *PLOS ONE* for specific examples. | The authors have declared that no competing interests exist. |

*Powered by Editorial Manager® and ProduXion Manager® from Aries Systems Corporation*

| | |
|---|---|
| **NO authors have competing interests**<br><br>Enter: *The authors have declared that no competing interests exist.*<br><br>**Authors with competing interests**<br><br>Enter competing interest details beginning with this statement:<br><br>*I have read the journal's policy and the authors of this manuscript have the following competing interests: [insert competing interests here]*<br><br>* typeset | |
| **Ethics Statement**<br><br>Enter an ethics statement for this submission. This statement is required if the study involved:<br><br>• Human participants<br>• Human specimens or tissue<br>• Vertebrate animals or cephalopods<br>• Vertebrate embryos or tissues<br>• Field research<br><br>Write "N/A" if the submission does not require an ethics statement.<br><br>General guidance is provided below. Consult the submission guidelines for detailed instructions. **Make sure that all information entered here is included in the Methods section of the manuscript.** | The specimens were obtained from either from markets or fishermen, or they were collected from the wild using a beach seine or hand net. The fish were then euthanized by immersion in buffered tricaine-methanesulphonate (MS 222) for thirty minutes after the animals stopped moving, in accordance with the guidelines outlined in the permits issued by Bangamata Sheikh Fojilatunnesa Mujib Science and Technology University's Ethics Committee (Letter no. 37.01.0044.064.05.001.24.553). |

Powered by Editorial Manager® and ProduXion Manager® from Aries Systems Corporation

| | |
|---|---|
| **Format for specific study types**<br><br>**Human Subject Research (involving human participants and/or tissue)**<br>• Give the name of the institutional review board or ethics committee that approved the study<br>• Include the approval number and/or a statement indicating approval of this research<br>• Indicate the form of consent obtained (written/oral) or the reason that consent was not obtained (e.g. the data were analyzed anonymously)<br><br>**Animal Research (involving vertebrate animals, embryos or tissues)**<br>• Provide the name of the Institutional Animal Care and Use Committee (IACUC) or other relevant ethics board that reviewed the study protocol, and indicate whether they approved this research or granted a formal waiver of ethical approval<br>• Include an approval number if one was obtained<br>• If the study involved *non-human primates*, add *additional details* about animal welfare and steps taken to ameliorate suffering<br>• If anesthesia, euthanasia, or any kind of animal sacrifice is part of the study, include briefly which substances and/or methods were applied<br><br>**Field Research**<br><br>Include the following details if this study involves the collection of plant, animal, or other materials from a natural setting:<br>• Field permit number<br>• Name of the institution or relevant body that granted permission | |
| **Data Availability**<br><br>Authors are required to make all data underlying the findings described fully available, without restriction, and from the time of publication. PLOS allows rare exceptions to address legal and ethical concerns. See the PLOS Data Policy and FAQ for detailed information. | No - some restrictions will apply |

| | |
|---|---|
| A Data Availability Statement describing where the data can be found is required at submission. Your answers to this question constitute the Data Availability Statement and **will be published in the article**, if accepted.<br><br>**Important:** Stating 'data available on request from the author' is not sufficient. If your data are only available upon request, select 'No' for the first question and explain your exceptional situation in the text box.<br><br>Do the authors confirm that all data underlying the findings described in their manuscript are fully available without restriction? | |
| **Describe where the data may be found in full sentences. If you are copying our sample text, replace any instances of XXX with the appropriate details.**<br><br>• If the data are **held or will be held in a public repository**, include URLs, accession numbers or DOIs. If this information will only be available after acceptance, indicate this by ticking the box below. For example: *All XXX files are available from the XXX database (accession number(s) XXX, XXX.*).<br>• If the data are all contained **within the manuscript and/or Supporting Information files**, enter the following: *All relevant data are within the manuscript and its Supporting Information files.*<br>• If neither of these applies but you are able to provide **details of access elsewhere**, with or without limitations, please do so. For example:<br><br>*Data cannot be shared publicly because of [XXX]. Data are available from the XXX Institutional Data Access / Ethics Committee (contact via XXX) for researchers who meet the criteria for access to confidential data.*<br><br>*The data underlying the results presented in the study are available from (include the name of the third party* | All relevant data are within the manuscript and its Supporting Information files. |

| | |
|---|---|
| *and contact information or URL).*<br>• This text is appropriate if the data are owned by a third party and authors do not have permission to share the data.<br><br>* typeset | |
| Additional data availability information: | |

Date: 28 August 2024

**Dear respected Sir, Editor of Journal of "PLOS ONE",**

I am sending our manuscript by Hasan et al. entitled "**Cryptic biodiversity of freshwater fish species in Bangladesh**" which is being submitted for possible publication in your Journal "Journal of PLOS ONE" by online portal. In this manuscript, all authors agreed to its submission and the Corresponding author has been authorized by co-authors. This manuscript has not been published before and is not concurrently being considered for publication elsewhere. This manuscript does not violate any copyright or other personal proprietary right of any person or entity and it contains no abusive, defamatory, obscene or fraudulent statements, nor any other statements that are unlawful in any way.

We suggest the following three potential referees with their email-addresses.


1. **Prof. Kazi Ahsan Habib, PhD**

Chairman
Department of Fisheries Biology and Genetics
Faculty of Fisheries, Aquaculture & Marine Science
Sher-e-Bangla Agricultural University (SAU)
Dhaka 1207, Bangladesh.
Phone: +88-02-44814069
Mobile: +880-1713682290
Email: habibka@sau.edu.bd
ahsan_sau@yahoo.com

2. **Dr. Mohammad Shafiqul Alam**

Associate Professor
Department of Genetics and Fish Breeding
Faculty of Fisheries
Bangabandhu Sheikh Mujibur Rahman Agricultural University
Gazipur, Bangladesh

Mobile: +880-1712718483
Email: msalamjp@gmail.com or msalam_fc@yahoo.com

3. **Prof. Dr. A.K.M. Rohul Amin**

Chairman
Department of Fisheries Biology and Genetics

Faculty of Fisheries, Hajee Mohammad Danesh Science and Technology University, Dinajpur, Bangladesh
Mobile: +880-1741261397
Email: akm_ramin@yahoo.com

I greatly appreciate for your kind arrangement for this manuscript. Correspondence regarding this manuscript should be sent to me addressed below. Thank you for your kind considerations on these matters. Best regards.

Sincerely yours,

Dr. Mahmudul Hasan
Assistant Professor
Department of Fisheries
Bangamata Sheikh Fojilatunnesa Mujib Science and Technology University
Jamalpur-2012.
Mobile: +8801714892205
Email: mhasan@bsfmstu.ac.bd/mhasan.fish@gmail.com
Website: http://www.mhasanbd.com/

Click here to access/download;Manuscript;Hasan_et_al.,_2024-

# Cryptic biodiversity of freshwater fish species in Bangladesh

Mahmudul Hasan[1,2], Chiaki Kambayashi[3], Zahid Hasan Anik[2] and Md. Saiful Islam[1]

[1]Department of Fisheries,Bangamata Sheikh Fojilatunnesa Mujib Science and Technology University, Jamalpur-2012, Bangladesh

[2]Evolution and Diversity Research Laboratory, Bangamata Sheikh Fojilatunnesa Mujib Science and Technology University, Jamalpur-2012, Bangladesh

[3]Faculty of Science, Niigata University,Niigata950-2181, Japan

*Corresponding author email: mhasan@bsfmstu.ac.bd, ORCID ID: https://orcid.org/0000-0002-7966-3426

Short title: Cryptic biodiversity of freshwater fish

## Abstract

Unrecognized cryptic species impede conservation planning and biodiversity assessments. DNA barcoding has tremendously expanded the number of novel species and cryptic species in biological science. Despite few sporadic studies, the exact number of freshwater species found in Bangladesh is not known. To assess this biodiversity, we sequenced the COI gene of 124freshwater specimens which were gathered from various locations around Bangladesh. Seven cryptic/candidate species hidden among the currently studied specimens were identified based on the findings of phylogenetic and species delimitation analyses that had included the data that was available for related species. Limited morphological examination was also applied to ensure the certainty of cryptic/candidate species. The appearance of new species, some of them possibly endemic has been hypothesized. This raises concerns regarding the true diversity and evolutionary history of freshwater species in Bangladesh, which are significantly underrepresented in the current systematic frameworks that do not account for DNA data. Our current study provides baseline data might aidlocal ichthyologists

in their quest to identify additional new species by combining several variables (morphology and ecology). Further research is warranted to protect the priceless freshwater species in Bangladesh.

Keywords: Cryptic species, freshwater fishes, conservation, Bangladesh

**INTRODUCTION**

Bangladesh is situated in the oriental region of the Indo-Himalayan and Indo-Chinese sub-regions[1]. This country is home to the Ganges-Brahmaputra river delta, which is the largest delta in the world. Approximately 230 rivers crisscross through the lands of Bangladesh. Among these rivers,57 are transboundary rivers (54 rivers are shared with India and three are shared with Myanmar)[2,3]. The downstream region consisting of many different rivers, canals, lakes with both flowing as well as static water (beels), and harbors is home to several freshwater fish. Approximately 265 species of freshwater fish are found in this lowland nation[4]. Freshwater fish are found in Bangladesh's biggest river systems, the Ganges, Brahmaputra, and Meghna, which are collectively equal to4,339,694 hectares of inland water[5]. Approximately 40% of all fish species found worldwide are freshwater fish and these are very valuable both nutritionally and economically[6]. The fisheries industry is unquestionably important for Bangladesh's economy, culture, and nutrition[7,8]. Additionally, the livelihoods of a sizable percentage of Bangladeshi population are closely associated with river fishing[9,10]. Majority of the fish produced in FY 2021–2022 amounted to 47.59 Lac mt[11], and were obtained from inland waters.

According to Shelley et al.[12], cryptic fish species present one of the main obstacles to the proper management, conservation, and planning of fish biodiversity. Cryptic species, which are genetic variants however, physically identical are a common occurrence. They are

concealed as nominal species. Accurate identification ofa species with the naked eye is difficult for ichthyologists as well, leading to errors in lists of species included in the literature. It is evident from several publications and review papers that, Bangladesh’s taxonomy is out-of-date and not harmonized, when compared with that of the neighboring countries[13]. Hence, an integrative taxonomy method incorporating molecular and morphological data is required to overcome this obstacle and provide a seamless and widely recognized list of species inhabiting this nation. Researchers describe additional new species of fish each year, but it is not clear as to how many species are endemic tothis nation.

In the current study, the authors have conducted phylogenetic analyses on fish specimens available in fish markets and natural resources nationwide to examine freshwater fish biodiversity in Bangladesh. Whenever necessary, morphological data has been used to identify cryptic or candidate species. The DNA-based barcoding method has proven to be an important molecular tool for non-specialists for species identification[14], and the mitochondrial cytochrome *c* subunit I (COI) sequence is a dependable barcode marker for measuring and assessing the taxonomic status of fish species[13,15,16,17]. This study attempts to review the biodiversity of Bangladesh's fisheries, and explore the possible cryptic species using the DNA barcoding technique.

## MATERIALS AND METHODS

### ***Study area, duration and identification of sample***

A total of124 fish specimens were gathered from all regions of Bangladesh (see Table 1). Their photographs and sexual orientation were recorded on the notepad. All specimens were received at the“Evolution and Diversity Research Laboratory” at Bangamata Sheikh Fojilatunnesa Mujib Science and Technology University, located in Jamalpur, Bangladesh. A voucher number was assigned to each specimen (please refer to Table 1). For subsequent

molecular research, the species weremaintained in either saturated DMSO/NaCl solution or 95% ethanol. Figure 1 lists the study sites in detail. Initial identification of the collected fish species was based on current taxonomy knowledge and literature dealing with the fisheries science[4]. If necessary, the names of the species names that have been accepted by the FishBase online portal have been adhered to[18].

**Fig 1. Map showing the different locations from where the specimens were collected.**

**Table 1: Specimens used and identified COI haplotypes found in this study**

| Voucher Number | Scientific Name | Local Name | Location | GPS | Sample Collection Date | Tissue Collection Date | Accession number |
|---|---|---|---|---|---|---|---|
| MHBSFMST U Fish 1 | *Cabdio morar* | Pieli | Sariakandi, Bogra | 24.919008,89.640545 | 01-07-2018 | 03-10-2022 | LC823243 |
| MHBSFMST U Fish 2 | *Tenualosailisha* | Ilish | Sariakandi, Bogra | 24.919008,89.640545 | 01-07-2018 | 03-10-2022 | LC823244 |
| MHBSFMST U Fish 3 | *Ailia sp.* | Bash patari/ Kajuli | Sariakandi, Bogra | 24.919008,89.640545 | 01-07-2018 | 03-10-2022 | LC823245 |
| MHBSFMST U Fish 5 | *Pisodonophisboro* | Bamush/ Kharu | Sariakandi, Bogra | 24.919008,89.640545 | 01-07-2018 | 03-10-2022 | LC823246 |
| MHBSFMST U Fish 6 | *Awaous sp.* | Bochabele | Sariakandi, Bogra | 24.919008,89.640545 | 01-07-2018 | 03-10-2022 | LC823247 |
| MHBSFMST U Fish 7 | *Mastacembelusarmatus* | ShalBaim | Sariakandi, Bogra | 24.919008,89.640545 | 01-07-2018 | 03-10-2022 | LC823248 |
| MHBSFMST U Fish 8 | *Botiadario* | Boumach | Sariakandi, Bogra | 24.919008,89.640545 | 01-07-2018 | 03-10-2022 | LC823249 |
| MHBSFMST U Fish 9 | *Channa orientalis* | Raga | Jaliarhaor, Netrokona | 24.692037,90.862910 | 25-08-2018 | 03-10-2022 | LC823250 |
| MHBSFMST U Fish 10 | *Aplocheilus panchax* | Kanpona | DangarBeel, Jamalpur | 24.918482,89.850998 | 02-02-2019 | 03-10-2022 | LC823251 |
| MHBSFMST U Fish 11 | *Trichogaster lalius* | Khoilsha | DangarBeel, Jamalpur | 24.918482,89.850998 | 02-02-2019 | 03-10-2022 | LC823252 |
| MHBSFMST U Fish 12 | *Trichogaster lalius* | Boro khoilsha | DangarBeel, Jamalpur | 24.918482,89.850998 | 02-02-2019 | 03-10-2022 | LC823253 |
| MHBSFMST U Fish 13 | *Trichogaster chuna* | Lal kholisha | DangarBeel, Jamalpur | 24.918482,89.850998 | 02-02-2019 | 03-10-2022 | LC823254 |
| MHBSFMST U Fish 14 | *Colisachuna* | Chuna Kholisha | DangarBeel, Jamalpur | 24.918482,89.850998 | 02-02-2019 | 03-10-2022 | LC823255 |
| MHBSFMST U Fish 15 | *Trichogaster chuna* | - | DangarBeel, Jamalpur | 24.918482,89.850998 | 02-02-2019 | 03-10-2022 | LC823256 |
| MHBSFMST U Fish 16 | *Macrognathus aral* | Tara Baim | Jaliarhaor, Netrokona | 24.692037,90.862910 | 25-08-2018 | 03-10-2022 | LC823257 |
| MHBSFMST U Fish 17 | *Lepidocephalichthys* sp. | Morichapui ya/ Gutum | Jaliarhaor, Netrokona | 24.692037,90.862910 | 25-08-2018 | 03-10-2022 | LC823258 |
| MHBSFMST U Fish 18 | *Ompok pabda* | Pabda | Gaglajur bazar, Netrokona | 24.876722,91.527330 | 25-08-2018 | 03-10-2022 | LC823259 |
| MHBSFMST U Fish 19 | *Mystus cavasius* | Gulsha | Jaliarhaor, Netrokona | 24.692037,90.862910 | 25-08-2018 | 03-10-2022 | LC823260 |
| MHBSFMST U Fish 20 | *Mystus tengara* | Tengra | Jaliarhaor, Netrokona | 24.692037,90.862910 | 25-08-2018 | 03-10-2022 | LC823261 |
| MHBSFMST U Fish 22 | *Mystus carcio* | Bujuri | Jaliarhaor, Netrokona | 24.692037,90.862910 | 25-08-2018 | 04-10-2022 | LC823262 |
| MHBSFMST U Fish 23 | *Channa marulius* | Gojar | Ghaglajur, Netrokona | 24.876722,91.527330 | 15-12-2018 | 04-10-2022 | LC823263 |
| MHBSFMST U Fish 25 | *Nandus nandus* | Bheda | Jaliarhaor, Netrokona | 24.692037,90.862910 | 25-08-2018 | 04-10-2022 | LC823264 |

| | | | | | | | |
|---|---|---|---|---|---|---|---|
| MHBSFMST U Fish 26 | *Channa punctata* | Taki | Jaliarhaor, Netrokona | 24.692037,90.862910 | 25-08-2018 | 04-10-2022 | LC823265 |
| MHBSFMST U Fish 27 | *Mystus cavasius* | Kabashi Tengra | Mawaghat Munshigonj | 23.466460,90.289461 | 25-08-2018 | 04-10-2022 | LC823266 |
| MHBSFMST U Fish 28 | *Bagarius bagarius* | Bagha air | Kongso, Netrokona | 24.880618,90.734221 | 25-08-2018 | 04-10-2022 | LC823267 |
| MHBSFMST U Fish 30 | *Sperata aorella* | Air | Kongso, Netrokona | 24.880618,90.734221 | 25-08-2018 | 04-10-2022 | LC823268 |
| MHBSFMST U Fish 31 | *Trichopsis vittata* | Poka Kholisha | MawaghatMunshigonj | 23.466460,90.289461 | 25-08-2018 | 04-10-2022 | LC823269 |
| MHBSFMST U Fish 32 | *Parambassis lala* | Lal chanda | Jaliarhaor, Netrokona | 24.692037,90.862910 | 25-08-2018 | 04-10-2022 | LC823270 |
| MHBSFMST U Fish 34 | *Xenentodon cancila* | Kakila | Jaliarhaor, Netrokona | 24.692037,90.862910 | 25-08-2018 | 04-10-2022 | LC823271 |
| MHBSFMST U Fish 36 | *Macrognathus pancalus* | Chikra/ Gujibaim | Jaliarhaor, Netrokona | 24.692037,90.862910 | 25-08-2018 | 04-10-2022 | LC823272 |
| MHBSFMST U Fish 37 | *Badis badis* | Napit koi | Jaliarhaor, Netrokona | 24.692037,90.862910 | 25-08-2018 | 04-10-2022 | LC823273 |
| MHBSFMST U Fish 38 | *Chaca chaca* | Bengachaca | Gaglajur Bazar, Netrokona | 24.876722,91.527330 | 25-08-2018 | 04-10-2022 | LC823274 |
| MHBSFMST U Fish 39 | *Notopterus notopterus* | Foli | Balikhola, Kishorganj | 24.224376,91.108948 | 25-08-2018 | 04-10-2022 | LC823275 |
| MHBSFMST U Fish 40 | *Labeo rohita* | Rui | Sokal bazar, Jamalpur | 24.929820,89.945901 | 17-04-2019 | 04-10-2022 | LC823276 |
| MHBSFMST U Fish 41 | *Labeo calbasu* | Kali baush | Sokal bazar, Jamalpur | 24.929820,89.945901 | 17-04-2019 | 04-10-2022 | LC823277 |
| MHBSFMST U Fish 46 | *Esomus danrica* | Darkina | Sokal bazar, Jamalpur | 24.929820,89.945901 | 25-08-2018 | 04-10-2022 | LC823278 |
| MHBSFMST U Fish 47 | *Setipinna phasa* | Chibuk chela/ Phasha | Guthail, Jamalpur | 25.087016,89.713601 | 25-08-2018 | 04-10-2022 | LC823279 |
| MHBSFMST U Fish 48 | *Catla catla* | Katol | Sokal bazar, Jamalpur | 24.929820,89.945901 | 17-04-2019 | 04-10-2022 | LC823280 |
| MHBSFMST U Fish 49 | *Cirrhinus mrigala* | Mrigal | Sanondobari, Jamalpur | 25.380929,89.744678 | 17-04-2019 | 04-10-2022 | LC823281 |
| MHBSFMST U Fish 50 | *Danio rerio* | Zebra anju/ Zebra | Kongsho, Sherpur | 24.997833,90.008218 | 17-04-2019 | 04-10-2022 | LC823282 |
| MHBSFMST U Fish 51 | *Corica soborna* | Subornakaski | Kongso, Sherpur | 24.997833,90.008218 | 15-03-2019 | 04-10-2022 | LC823283 |
| MHBSFMST U Fish 52 | *Salmostoma phulo* | Narikeli Chela/ Katari | Sokal bazar, Jamalpur | 24.929820,89.945901 | 02-02-2019 | 04-10-2022 | LC823284 |
| MHBSFMST U Fish 53 | *Hyporhamphus limbatus* | Ekthota | Kendua, Netrokona | 24.66386,90.84131 | 15-03-2019 | 04-10-2022 | LC823285 |
| MHBSFMST U Fish 54 | *Channa striata* | Shol | Kendua, Netrokona | 24.66386,90.84131 | 02-02-2019 | 04-10-2022 | LC823286 |
| MHBSFMST U Fish 55 | *Pseudeutropius atherinoides* | Batai | Kendua, Netrokona | 24.66386,90.84131 | 22-04-2019 | 04-10-2022 | LC823287 |
| MHBSFMST U Fish 56 | *Ophichthys* sp. | kuchia | Sokal bazar, Jamalpur | 24.929820,89.945901 | 02-02-2019 | 04-10-2022 | LC823288 |
| MHBSFMST U Fish 57 | *Wallago attu* | Boal | Kongso, Netrokona | 24.880618,90.734221 | 25-08-2018 | 04-10-2022 | LC823289 |
| MHBSFMST U Fish 59 | *Heteropneustes fossilis* | Shing | Dangarbeel, Jamalpur | 24.918482,89.850998 | 02-02-2019 | 04-10-2022 | LC823290 |
| MHBSFMST U Fish 61 | *Puntius sophore* | Jatpunti/ Bhadiputi | JaliarHaor, Netrokona | 24.692037,90.862910 | 25-08-2018 | 04-10-2022 | LC823291 |
| MHBSFMST U Fish 63 | *Pethia conchonius* | Tit punti | Sokal bazar, Jamalpur | 24.929820,89.945901 | 17-04-2019 | 04-10-2022 | LC823292 |
| MHBSFMST U Fish 64 | *Chanda nama* | Chanda (lomba) | Balikhula, Kishorgonj | 24.224376,91.108948 | 17-04-2019 | 04-10-2022 | LC823293 |
| MHBSFMST U Fish 65 | *Parambassis ranga* | Chanda (Gol)/ Phopa | Kendua, Netrokona | 24.66386,90.84131 | 17-04-2019 | 04-10-2022 | LC823294 |
| MHBSFMST U Fish 66 | *Macrognathus pancalus* | Guchibaim | Mawaghat, Munshigonj | 23.466460,90.289461 | 17-04-2019 | 04-10-2022 | LC823295 |
| MHBSFMST U Fish 67 | *Amblypharyngodon mola* | Fakasemola | JaliarHaor, Netrokona | 24.692037,90.862910 | 17-04-2019 | 04-10-2022 | LC823296 |
| MHBSFMST U Fish 69 | *Channa gachua* | Cheng | Dangarbeel, Jamalpur | 24.918482,89.850998 | 17-04-2019 | 04-10-2022 | LC823297 |
| MHBSFMST U Fish 70 | *Salmostoma bacalia* | Ful chela | Sokal bazar, Jamalpur | 24.929820,89.945901 | 17-04-2019 | 04-10-2022 | LC823298 |
| MHBSFMST | *Eutropiichthys vacha* | Bacha | Ghaglajur, | 24.876722,91.527330 | 25-08-2018 | 04-10-2022 | LC823299 |

| | | | | | | | |
|---|---|---|---|---|---|---|---|
| U Fish 71 | | | Netrokona | | | | |
| MHBSFMST U Fish 72 | *Gudusia chapra* | GoniChapil a | Sariakandi, Bogra | 24.919008,89.640545 | 01-07-2018 | 04-10-2022 | LC823300 |
| MHBSFMST U Fish 73 | *Clupisoma garua* | Ghaura | Mawaghat, Munshigonj | 23.466460,90.289461 | 17-04-2019 | 04-10-2022 | LC823301 |
| MHBSFMST U Fish 74 | *Rita rita* | Rita | Balikhola, Kishorgonj | 24.224376,91.108948 | 01-07-2018 | 04-10-2022 | LC823302 |
| MHBSFMST U Fish 75 | *Hemibagrus menoda* | Hugli/ Arwari/ Ghagla | Ghaglajur bazaar, Netrokona | 24.876722,91.527330 | 01-07-2018 | 04-10-2022 | LC823303 |
| MHBSFMST U Fish 77 | *Leiodon cutcutia* | Patipotka | Bramaputra river, Jamalpur | 24.926939,89.961839 | 10-11-2018 | 04-10-2022 | LC823304 |
| MHBSFMST U Fish 78 | *Devario devario* | Hingra kata punti/ Debari/ Chapchela | Ghaglajur Bazar, Netrokona | 24.876722,91.527330 | 02-02-2019 | 04-10-2022 | LC823305 |
| MHBSFMST U Fish 79 | *Osteobrama cotio* | Dhela | Gaglajur Bazar, Netrokona | 24.876722,91.527330 | 02-02-2019 | 04-10-2022 | LC823306 |
| MHBSFMST U Fish 80 | *Mylopharyngodon piceus* | Mohashol | Sokal Bazar, Jamalpur | 24.929820,89.945901 | 25-08-2019 | 04-10-2022 | LC823307 |
| MHBSFMST U Fish 81 | *Labeo gonius* | Gonia | Sokal Bazar, Jamalpur | 24.929820,89.945901 | 25-08-2019 | 04-10-2022 | LC823308 |
| MHBSFMST U Fish 82 | *Labeo gonius* | Tatkini/ Bhagna/ Raik/ Lacho/bata | Balikhula, Kishorgonj | 24.224376,91.108948 | 25-08-2019 | 12-10-2022 | LC823309 |
| MHBSFMST U Fish 83 | *Erethistes sp.* | Kutakanti | Ghaglajur Bazar, Netrokona | 24.876722,91.527330 | 25-08-2019 | 12-10-2022 | LC823310 |
| MHBSFMST U Fish 84 | *Gagata youssoufi* | Gum/ Gagata | Ghaglajur, Netrokona | 24.876722,91.527330 | 25-08-2019 | 12-10-2022 | LC823311 |
| MHBSFMST U Fish 85 | *Pethia gelius* | Gilipunti/ puti | Ghaglajur Bazar, Netrokona | 24.876722,91.527330 | 07-01-2020 | 12-10-2022 | LC823312 |
| MHBSFMST U Fish 86 | *Rhinomugil corsula* | Folla, khalla, halla, khorsula | Balikhula, Kishorgonj | 24.224376,91.108948 | 01-01-2020 | 12-10-2022 | LC823313 |
| MHBSFMST U Fish 87 | *Chandramara chandramara* | Jolbujuri | Ghaglajur Bazar, Netrokona | 24.876722,91.527330 | 07-01-2020 | 12-10-2022 | LC823314 |
| MHBSFMST U Fish 90 | *Silonia Silondia* | Shilong | Kawniar char, Dewangonj | 25.399803,89.796954 | 15-01-2020 | 12-10-2022 | LC823315 |
| MHBSFMST U Fish 91 | *Glyptothorax telchitta* | Lalkutakant i | Kawniar char, Dewangonj | 25.399803,89.796954 | 15-01-2020 | 12-10-2022 | LC823316 |
| MHBSFMST U Fish 92 | *Gagata cenia* | Kutakanti, Cenia, Jangla, Kaowa, Gang tengra | Sanondabar, Jamalpur | 25.380929,89.744678 | 25-01-2020 | 12-10-2022 | LC823317 |
| MHBSFMST U Fish 93 | *Gogangra laevis* | Gang Tengra | Dewangonj bazar, Jamalpur | 25.167125,89.765972 | 15-01-2020 | 12-10-2022 | LC823318 |
| MHBSFMST U Fish 96 | *Rasbora daniconius* | Darkina | Kendua, Netrokna | 24.66386,90.84131 | 22-01-2020 | 12-10-2022 | LC823319 |
| MHBSFMST U Fish 97 | *Botia lohachata* | Bou/Rani | Sokal bazar, Jamalpur | 24.929820,89.945901 | 22-01-2020 | 12-10-2022 | LC823320 |
| MHBSFMST U Fish 98 | *Labeo boggut* | Vangon/ Gonari | Sokal bazar, Jamalpur | 24.929820,89.945901 | 22-01-2020 | 12-10-2022 | LC823321 |
| MHBSFMST U Fish 99 | *Paracanthocobitis abutwebi* | Balichata/ Puiya | Sanondabari, Jamalpur | 25.380929,89.744678 | 25-01-2020 | 12-10-2022 | LC823322 |
| MHBSFMST U Fish 100 | *Schistura sp.* | Balikhura/ Puiya | Sanondabari, Jamalpur | 25.380929,89.744678 | 25-01-2020 | 12-10-2022 | LC823323 |
| MHBSFMST U Fish 101 | *Anodontostomachacunda* | Chacunda | Defla Bazar, Jamalpur | 25.021425,89.837869 | 25-01-2020 | 12-10-2022 | LC823324 |
| MHBSFMST U Fish 102 | *Salmostoma bacalia* | Chela | Soal bazar, Jamalpur | 24.929820,89.945901 | 21-12-2020 | 12-10-2022 | LC823325 |
| MHBSFMST U Fish 103 | *Badis badis* | Napit koi | Sokal bazar, Jamalpur | 24.929820,89.945901 | 01-02-2020 | 12-10-2022 | LC823326 |
| MHBSFMST U Fish 104 | *Rhinomugil corsula* | Flathead mullet | Sokal Bazar, Jamalpur | 24.929820,89.945901 | 01-02-2020 | 12-10-2022 | LC823327 |
| MHBSFMST | *Pethia conchonius* | Kanchonput | Guthail, | 25.087016,89.713601 | 05-02-2020 | 12-10-2022 | LC823328 |

| | | | | | | | |
|---|---|---|---|---|---|---|---|
| U Fish 105 | | i/ Taka puti | Jamalpur | | | | |
| MHBSFMST U Fish 106 | *Neoeucirrhichthys maydelli* | GualParapui ya | Guthail, Jamalpur | 25.087016,89.713601 | 05-02-2020 | 12-10-2022 | LC823329 |
| MHBSFMST U Fish 107 | *Chela laubuca* | Chep chela | Guthail, Jamalpur | 25.087016,89.713601 | 05-02-2020 | 12-10-2022 | LC823330 |
| MHBSFMST U Fish 108 | *Tariqilabeo latius* | Matikhora/k alabata | Guthail, Jamalpur | 25.087016,89.713601 | 05-02-2020 | 12-10-2022 | LC823331 |
| MHBSFMST U Fish 109 | *Pangio Pangia* | Kuttapuiya/ Panga/ Kolipuiya | Guthail, Jamalpur | 25.087016,89.713601 | 05-02-2020 | 12-10-2022 | LC823332 |
| MHBSFMST U Fish 110 | *Odontamblyopus rubicundus* | Lalchewa | Mawaghat, Munshigonj | 23.466460,90.289461 | 08-01-2020 | 12-10-2022 | LC823333 |
| MHBSFMST U Fish 111 | *Apocryptes bato* | Chewa/ Chiring | Mawaghat, Munshigonj | 23.466460,90.289461 | 08-01-2020 | 12-10-2022 | LC823334 |
| MHBSFMST U Fish 112 | *Sperata aor* | Guizza air | Mawaghat, Munshigonj | 23.466460,90.289461 | 08-01-2020 | 12-10-2022 | LC823335 |
| MHBSFMST U Fish 118 | *Ompok bimaculatus* | Kala pabda | Rangamati | 22.966884,92.201557 | 11-01-2020 | 13-10-2022 | LC823336 |
| MHBSFMST U Fish 121 | *Lates calcarifer* | Deshivetki | PaikariMotsho Arot, Barishal | 22.703728,90.375656 | 27-01-2021 | 13-10-2022 | LC823337 |
| MHBSFMST U Fish 126 | *Cynoglossus sp.* | Kukurjib | Fishery Ghat, Chittagong | 22.328864,91.847110 | 11-02-2021 | 13-10-2022 | LC823338 |
| MHBSFMST U Fish 130 | *Polynemus paradiseus* | Taposhi | PaikariMotsho Arot, Barishal | 22.703728,90.375656 | 27-01-2021 | 13-10-2022 | LC823339 |
| MHBSFMST U Fish 134 | *Tariqilabeo latius* | Kalobata | Shampurnagar ghat, Rajshahi | 24.348801,88.657363 | 22-12-2020 | 13-10-2022 | LC823340 |
| MHBSFMST U Fish 135 | *Psilorhynchus sucatio* | Titari | Charlokkhipur Shibgonj, Chapainawabg onj | 24.598931,88.088913 | 21-12-2020 | 13-10-2022 | LC823341 |
| MHBSFMST U Fish 136 | *Glossogobius giuris* | Bele | Narayanpur, Shibgonj, Chapainawabg onj | 24.568242,88.096875 | 21-12-2020 | 13-10-2022 | LC823342 |
| MHBSFMST U Fish 137 | *Setipinna phasa* | Pasha | Charlokkhipur , Shibgonj, Chapai Nawabgonj | 24.598931,88.088913 | 21-12-2020 | 13-10-2022 | LC823343 |
| MHBSFMST U Fish 138 | *Terapon jarbua* | Rekha | Shondhya bazar, Moylaputa,Kh ulna | 22.812974,89.557110 | 28-01-2021 | 13-10-2022 | LC823344 |
| MHBSFMST U Fish 139 | *Cynoglossus sp.* | Kukurjib | RupshaGhat, Khulna | 22.801526,89.581146 | 28-01-2021 | 13-10-2022 | LC823345 |
| MHBSFMST U Fish 140 | *Maculabatis pastinacoides* | Shapla pata | Paikarimothso Arot, Barishal | 22.703728,90.375656 | 27-01-2021 | 13-10-2022 | LC823346 |
| MHBSFMST U Fish 141 | *Stigmatogobius sadanandio* | Nondibaila | RupshaGhat, Khulna | 22.807220,89.580416 | 28-01-2021 | 13-10-2022 | LC823347 |
| MHBSFMST U Fish 142 | *Acentrogobius viridipunctatus* | Shobujfutki baila | Sondha bazar, Rupsha, Khulna | 22.813079,89.556941 | 28-01-2021 | 13-10-2022 | LC823348 |
| MHBSFMST U Fish 143 | *Apocryptes bato* | Chiring | Sondha bazar, Rupsha, Khulna | 22.813079,89.556941 | 28-01-2021 | 13-10-2022 | LC823349 |
| MHBSFMST U Fish 145 | *Trypauchen vagina* | Raja chewa | RupshaGhat, Khulna | 22.801526,89.581142 | 28-01-2021 | 13-10-2022 | LC823350 |
| MHBSFMST U Fish 146 | *Platycephalus indicus* | Mur baila | RupshaGhat, Khulna | 22.801526,89.581142 | 28-01-2021 | 13-10-2022 | LC823351 |
| MHBSFMST U Fish 147 | *Eleutheronema tetradactylum* | Tailla | RupshaGhat, Khulna | 22.801526,89.581142 | 28-01-2021 | 13-10-2022 | LC823352 |
| MHBSFMST U Fish 148 | *Mystus gulio* | Nunatengra | Paikarimothso Arot,Barishal | 22.703728,90.375656 | 27-01-2021 | 13-10-2022 | LC823353 |
| MHBSFMST U Fish 149 | *Sillaginopsis domina* | Kulardati/ Tulardanti | Paikarimothso Arot, Barishal | 22.703728,90.375656 | 27-01-2021 | 13-10-2022 | LC823354 |
| MHBSFMST U Fish 150 | *Toxotes chatareus* | Deha | RupshaGhat, Khulna | 22.801526,89.581142 | 25-01-2021 | 13-10-2022 | LC823355 |
| MHBSFMST U Fish 151 | *Arius gagora* | Jonglitengra | RupshaGhat, Khulna | 22.801526,89.581142 | 28-01-2021 | 13-10-2022 | LC823356 |
| MHF154 | *Puntius sophore* | Chela puti | HaziShariotull ah Market, Faridpur | 23.601208,89.830870 | 26-01-2021 | 13-10-2022 | LC823357 |
| MHBSFMST U Fish 156 | *Gagata gagata* | Gang tengra/ Deshitengra | PaikariMotsho Arot, Barishal | 22.703728,90.375656 | 27-01-2021 | 13-10-2022 | LC823358 |

| | | | | | | | |
|---|---|---|---|---|---|---|---|
| MHBSFMSTU Fish 157 | *Awaous sp.* | Chewa/Bele | Tepakhula Bazar, Faidpur | 23.613323,89.856330 | 26-01-2021 | 13-10-2022 | LC823359 |
| MHBSFMSTU Fish 158 | *Glossogobius giuris* | Lalbele | Tepakhula Bazar, Faidpur | 23.613323,89.856330 | 26-01-2021 | 13-10-2022 | LC823360 |
| MHBSFMSTU Fish 159 | *Glossogobius giuris* | Bele | Tepakhula Bazar, Faidpur | 23.613323,89.856330 | 26-01-2021 | 13-10-2022 | LC823361 |
| MHBSFMSTU Fish 161 | *Acanthopagrus latus* | Datina | Chakaria, Chittagong | 21.751155,92.019712 | 26-01-2021 | 13-10-2022 | LC823362 |
| MHBSFMSTU Fish 163 | *Coilia ramcarati* | Holudolua | Chakaria, Chittagong | 21.751155,92.019712 | 27-01-2021 | 13-10-2022 | LC823363 |
| MHBSFMSTU Fish 164 | *Nandus nandus* | Meni | Ilisharot, Rangamati | 22.648545,92.184714 | 30-01-2021 | 13-10-2022 | LC823364 |
| MHBSFMSTU Fish 301 | *Amblypharyngodon mola* | Lalmola | Mawaghat, Munshigonj | 23.466460,90.289461 | 08-01-2020 | 13-10-2022 | LC823365 |
| MHBSFMSTU Fish 302 | *Awaous sp.* | Bele Mach | Tepakhula Bazar, Faidpur | 23.613323,89.856330 | 26-01-2021 | 13-10-2022 | LC823366 |

### *DNA extraction and sequencing*

Whole DNA of all fish specimens was extracted and purified from conserved fin tissues using Chelex 100[19]. Using the primers Fish-F1, Fish-F2, Fish-R1, and Fish-R2, the partial COI fragment was amplified by polymerase chain reaction (PCR) using EmeraldAmp PCR Master Mix (Takara Bio)[20]. Cycle conditions described in a previously conducted study[13] were followed for performing PCR. The amplified PCR products were purified using polyethylene glycol precipitation. Cycle sequencing procedures were then performed using the BigDye Terminator v3.1 Cycle Sequencing Kit (Applied Bioscience). Sequencing was performed using ABI 3500 automated sequencers. The newly discovered sequences were incorporated inthe International Nucleotide Sequence Databases (INSD) through the DNA Data Bank of Japan (Accession numbers LC823243–LC823366).

### *Phylogenetical analysis*

The resulting COI gene nucleotide sequences were matched with additional COI sequences of Bangladeshi fish from INSD[13] and the MAFFT(Multiple Alignment using Fast Fourier Transform)v. 7.427 with the L-INS-I option[21]. Phylogenetic trees were inferred using maximum likelihood (ML) and Bayesian inference (BI) techniques. IQ-TREE v. 1.6.12 was used to estimate nucleotide substitution models for ML and BI analyses using AICc[22]. The

IQ-TREE was used to determine the ML phylogeny, and 1000 pseudo replicates were used for an ultra-fast bootstrapping (BS). MrBayes v. 3.2.6 was used to obtain the Bayesian posterior probabilities and BI tree[23].A tree sample was obtained after every 1000 generations, throughout two separate runs of four Markov chains totaling 100 million generations. Tracer v. 1.7.1[24] was used to examine the parameter estimates and convergence, and the first 10%trees were eliminated in light of the findings. MEGA11's pairwise-deletion option was used for computing pairwise comparisons of the uncorrected p-distance[25] (**S1 Table).**

***Species delimitation analyses***

Species delimitation investigations were conducted using Assemble Species by Automatic Partitioning (ASAP)[26] based on712 COI sequences used in phylogenetic analysis **(S2 Table)**. The ASAP analysis was conducted using the online ASAP version (https:// bioinfo.mnhn.fr/abi/public/asap/asapweb.html) using the uncorrected pairwise distances with the default parameters.

## RESULTS

Fish specimens were gathered nationwide from more than 30 locations (Fig 1), and the mitochondrial COI sequences of 124 individuals were determined. Sequences with ≥1 nucleotide change were considered distinct haplotypes. Our COI alignment matrix consisted of 655 bp representing 119 haplotypes. Table 1 provides a summary of all haplotypes. Based on COI sequences, nearly identical topologies were observed in the ML (ln $L$ = −59788.54) and BI (ln $L$ = −60808.92; not shown) trees (**S1 Fig.)**.

Uncorrected p-distance and species delimitation analysis demonstrated 243 groups with the best score . Most of the OTUs (operational taxonomy units) sequenced in this study were assigned to groups with known species; however, some specimens formed their own unique groups. In particular, seven OTUs had to be assigned to different groups, despite the presence of the same species identified morphologically, indicating that these specimens were cryptic species **(S3 Table)**.

Specifically, the specimen of MHBSFMSTU Fish 3 had been morphologically identified as *Ailia colia*, but it was observed to belong to a different species (*Ailia* sp.) compared to its nearest congener (COI divergence 8.2%) (Fig 2B). This specimen was collected from the area adjacent to the Jamuna River (Sariakandi, Bogura). The specimens 157 and 302 of MHBSFMSTU Fish 6, formed clades with *Awaous* sp. and demonstrated poor degrees of divergence (COI divergence 0.3%) (Fig 2C). These specimens were collected from the Bogura and Faridpur districts which are linked with the rivers Jamuna and Padma. MHBSFMSTU Fish 17 identified as *Lepidocephalichthys* sp. collected from Netrokona (24.692037,90.862910), formed clades with other known sequences (MK572291, MK572294)and demonstrated15.50% COI divergence from its nearest congeners (Fig 2B). The specimen MHBSFMSTU Fish 56, assigned as *Ophichthys* sp.demonstrated 7.7% genetic divergence from its near congeners (Fig 2B).The specimen MHBSFMSTU Fish 83, identified as *Erethistes* sp. belonging to the genus *Erethistes*, exhibited a genetic variation of more than 5.9% from the most closely related *E. pusilus* (Fig 2C). This specimen was collected from the Netrokona region. Similarly, the specimen MHBSFMSTU Fish 100, identified as *Schuster* sp. (collected from Sakal Bazar, Jamalpur), belonging to the genus *Schistura* demonstrated a genetic variation of more than 9.2% from its closest related species *Schuster corica* (Fig 2A). Two specimens (MHBSFMSTU Fish 126 and 139), collected from Khulna and Chittagong close to the coastal belt in Bangladesh, identified as *Cynoglossus* sp. were found to be

distantly related (COI gene divergence 17.52%) with their congeners *Cynoglossus* (MK572284) (Fig 2D).

**Fig 2A. Maximum Likelihood (ML) tree of freshwater species of Bangladesh based on nucleotide sequences of COI gene (partial portion of S1 Fig.).**

**Fig 2B. Maximum Likelihood (ML) tree of freshwater species of Bangladesh based on nucleotide sequences of COI gene (partial portion of S1 Fig.).**

**Fig 2C. Maximum Likelihood (ML) tree of freshwater species of Bangladesh based on nucleotide sequences of COI gene (partial portion of S1 Fig.).**

**Fig 2D. Maximum Likelihood (ML) tree of freshwater species of Bangladesh based on nucleotide sequences of COI gene (partial portion of S1 Fig.).**

## DISCUSSION

Our research identified seven cryptic fish species in the northeastern areas of Bangladesh and has contributed to a more thorough method of clarifying species delimitation based onCOI gene concentration. Although Rahman et al.[13]developedthe DNA barcoding system, our work largely differs from their workwith few overlapping aspects due to the study's inadequate coverage of Bangladesh's western and southwestern regions, primarily the Sundarbans and a section of river Ganga and its tributaries. We attempted to cover the Sherpurregionand some selected areas of the Mymensingh region as well as the exclusive regionof the Old Brahmaputra River, which is included in the Ganga tributaries (see Fig 1). Although specimens from the Khulna (Sundarban) area are limited, we have included samples from this location (see Table1). They were able identify several COI sequences and connect them to previously identified species, but they have recommended that the most significant aspect of the DNA barcoding is that the end users can access it and can alter taxonomies to help with identification, nomenclature, species delimitation, introgression, and multiple mitochondrial

lineages. However, it appears that this was only the beginning of the barcoding effort. As a result, this study is an extra effort to produce more DNA barcode data and facilitate specimen identification. A major portion of Bangladesh's small indigenous species is a primary source of protein, and in the long run, DNA barcoding might enable their identification and appropriate management for conservation. However, our goal was creating a small-scale DNA barcode library to assist local taxonomists who can utilize this information as a reference and to identify and differentiate between real, cryptic, and candidate species. This information is often used for nomenclature revision, taxonomy revision, and the identification of any distinct lineages or introgressions that may exist somewhat apart from their nominal species.

Based on the results of this biodiversity study, it is tempting to make conjectures about the extent of unexplored freshwater fish diversity that still exists in Bangladesh. There still might exist several undiscovered cryptic species in Bangladesh. Our investigation involving the analysis of the COI gene has revealed the presence of seven likely cryptic species. These species are genetically distinct from their closely related counterparts. *Ailia* sp. (MHBSFMSTU Fish 3) is often wrongly identified as its closely related species, *Ailiacoila*. Our morphological and genetic findings indicate that *Ailia* sp. is cryptic and necessitates further investigation, utilizing multiple datasets, to establish its classification as a new species. Other genera, such as *Awaous*, *Lepidocephalichthys*, *Ophichthys*, *Erethistes*, *Schistura*, and *Cynoglossus*, contain cryptic species as well. Our ongoing research canformally identify these seven enigmatic species as new species. The number of recognized species in Bangladesh can increase, and it is furthernecessary to investigate the type site of each of these selected cryptic species in order to clarify their taxonomic status. Despite being a low-lying country, Bangladesh’s robust river systems can play a significant role in the process of speciation. The occurrence of cryptic, spatially dictated variety in fish species in

Bangladesh, as indicated in this study, is not a recent revelation. Hasan et al.[2] and Raman et al.[13] have reported a comparable pattern for frogs and fish in Bangladesh, respectively. The presence of several overlooked species of freshwater bony fishes, such as loach and gobi, in this study indicates that the hidden diversity among well-known and widely distributed fish species in Bangladesh might be more prevalent than previously believed. Additionally, exclusive studies on the complete river systems of Bangladesh, which include the Padma, Meghna, and Jamuna rivers are warranted. The findings given here possibly reveal only a small portion of the overall freshwater fish biodiversity in the region.

## CONCLUSION

Managing the biodiversity of any fishery requires the identification of the species in it. Contradictory to previous research, the integrative taxonomy technique offers a fresh perspective on the hidden biodiversity in Bangladesh. Some researchers have recently been using some molecular technologies, but their efforts still trail behind those of the neighboring countries. Above all, annotated species lists are essential for creating appropriate conservation policies. Our findings of seven cryptic species **(S3 Table)** provide additional information regarding the biodiversity in the fisheries in Bangladesh. Future research including more exclusive sampling and quantification of the obtained data by utilizing integrative taxonomy approach (morphology, molecular and ecology) can further expand the list of species in Bangladesh. Researchers and policymakers can utilize the results of our study, which reveals that Bangladesh is home to many cryptic species, as baseline data. As a result, the study project we have in mind can greatly advance our understanding of the biodiversity in Bangladesh's fisheries and aid in the formulation of sound conservation guidelines.

**Ethics Statement**

The specimens were obtained from either from markets or fishermen, or they were collected from the wild using a beach seine or hand net. The fish were then euthanized by immersion in buffered tricaine-methanesulphonate (MS 222) for thirty minutes after the animals stopped moving, in accordance with the guidelines outlined in the permits issued by Bangamata Sheikh Fojilatunnesa Mujib Science and Technology University's Ethics Committee (Letter no. 37.01.0044.064.05.001.24.553).

**Data Availability**

The INSD provides all sequence and related voucher data. Supplementary Information contains voucher metadata.

**ACKNOWLEDGEMENT**

Authors are thankful to Ministry of Science and Technology, the People's Republic of Bangladesh for funding. We are grateful to Associate Professor, Nagahama Institute of Bio-Science and Technology for some technical support to accomplish the molecular work.

**AUTHOR CONTRIBUTIONS**

Conceptualization: Mahmudul Hasan, Data curation: Chiaki Kambayashi, Formal analysis: Chiaki Kambayashi. Funding acquisition: Mahmudul Hasan, Md. Saiful Islam. Investigation: Mahmudul Hasan, Methodology: Mahmudul Hasan, Zahid Hasan Anik. Supervision: Mahmudul Hasan. Writing – original draft: Mahmudul Hasan, Chiaki Kambayashi. Writing – review & editing: Mahmudul Hasan, Chiaki Kambayashi.

## REFERENCES


1. Nishat A, Huq SMI, Barua SP, Reza AHMA, Khan ASM. Bio-Ecological Zones of Bangladesh. IUCN, The World Conservation Union, Bangladesh Country Office. 141 pp. 2002.
2. Hasan M, Islam MM, Khan MMR, Alam MS, Kurabayashi A, Igawa T, Kuramoto M, Sumida M. Cryptic anuran biodiversity in Bangladesh revealed by mitochondrial 16S rRNA gene sequences. Zoological Science. 2012; 29:162–172.
3. Afroz R, Rahman MA. Transboundary river water for Ganges and Teesta Rivers in Bangladesh: an assessment. Global Science and Technology Journal. 2013; 1(1):100–111.
4. Rahman AKA. Freshwater Fishes of Bangladesh, Second Edition. Zoological Society of Bangladesh, Department of Zoology, University of Dhaka. 394 pp. 2005.
5. Hussain MG, Mazid MA. Genetic improvement and conservation of carp species in Bangladesh. Bangladesh Fisheries Research Institute and International Center for Living Aquatic Resources Management. 74 pp. 2001.
6. Ghorbani R, Abbasi F, Molaei M, Naeimi A. Identification and distribution of fish fauna in Kaboodval Stream (Golestan Province, Iran). World Journal of Fish and Marine Sciences. 2013; 5(5):467–473.
7. Hossain MAR, Wahab MA, Belton B. The checklist of the riverine fishes of Bangladesh. FAN Bangladesh. 2012; 2:29–39.
8. Hasan M, Islam MS. Collection, preservation and identification of freshwater fish species in middle to north-east Bangladesh with special notes on phenotypic plasticity of few species. World Journal of Biology and Biotechnology. 2022; 7(2):7–13.

9. Hasan M, Tripti SMI. Biodiversity, threats and recommendation for conservation of fishes in the Old Brahmaputra River. Bangladesh Journal of Fisheries. 2021; 33(2):347–354.
10. Hasan M, Anik H, Kurabayashi A. Assessment of fish biodiversity in the Padma river from Farakka Barrage point to the Shampur Nagar Ghat, Rajshahi, Bangladesh. Bangladesh Journal of Fisheries. 2023; 35(1):13–23.
11. Department of Fisheries. National Fish Week 2023 Compendium (in Bangla). Department of Fisheries, Ministry of Fisheries and Livestock, Bangladesh. 160p. 2023.
12. Shelley JJ, Swearer SE, Adams M, Dempster T, Feuvre MCL, Hammer MP, Unmack PJ. Cryptic biodiversity in the freshwater fishes of the Kimberley endemism hotspot, northwestern Australia. Molecular Phylogenetics and Evolution. 2018; 127:843–858.
13. Rahman MM, Norén M, Mollah AR, Kullander SO. Building a DNA barcode library for the freshwater fishes of Bangladesh. Scientific Reports. 2019; 9:9382.
14. Hebert PD, Cywinska A, Ball SL, deWaard JR. Biological identifications through DNA barcodes. Proceedings of the Royal Society of London B: Biological Sciences. 2003; 270(1512):313–321.
15. Lakra WS, Singh M, Goswami M, Gopalakrishnan A, Lal KK, Mohindra V, Sarkar UK, Punia PP, Singh KV, Bhatt JP Ayyappan S. DNA barcoding Indian freshwater fishes, Mitochondrial DNA Part A. 2016; 27:4510–4517.
16. Habib KA, Islam MJ, Sakib MN, Brishti PS, Neogi AK. DNA barcoding of reef-associated fishes of Saint Martin's Island, Northern Bay of Bengal, Bangladesh. Ecology and Evolution. 2023; 13:e10641.
17. Habib KA, Neogi AK, Rahman M, Oh J, Lee Y-H, Kim C-G. DNA barcoding of brackish and marine water fishes and shellfishes of Sundarbans, the world’s largest mangrove ecosystem. PLoS ONE. 2021; 16(8):e0255110.

18. Froese R, Pauly D. FishBase. World Wide Web electronic publication. www.fishbase.org, version (02/2024). 2024.
19. Walsh PS, Metzger DA, Higuchi R. Chelex 100 as a medium for simple extraction of DNA for PCR-based typing from forensic material. Biotechniques. 1991; 10(4):506–513.
20. Ward RD, Zemlak TS, Innes BH, Last PR, Hebert PDN. DNA barcoding Australia's fish species. Philosophical Transactions of The Royal Society B Biological Sciences. 2005; 360:1847–1857.
21. Katoh K, Standley DM. MAFFT multiple sequence alignment software version 7: improvements in performance and usability. Molecular Biology and Evolution. 2013; 30:772–780.
22. Nguyen L-T, Schmidt HA, von Haeseler A and Minh BQ. IQ-TREE: a fast and effective stochastic algorithm for estimating maximum-likelihood phylogenies. Molecular Biology and Evolution. 2015; 32:268–274.
23. Ronquist F, Teslenko M, Van Der Mark P, Ayres DL, Darling A, Höhna S, Larget B, Liu L, Suchard MA, Huelsenbeck JP. MrBayes 3.2: efficient Bayesian phylogenetic inference and model choice across a large model space. Systematic Biology. 2012; 61:539–542.
24. Rambaut A, Drummond AJ, Xie D, Baele G, Suchard MA. Posterior summarization in Bayesian phylogenetics using Tracer 1.7. Systematic Biology. 2018; 67:901–904.
25. Tamura K, Stecher G, Kumar S. MEGA11: molecular evolutionary genetics analysis version 11. Molecular Biology and Evolution. 2021; 38:3022–3027.
26. Puillandre N, Brouillet S, Achaz G. ASAP: assemble species by automatic partitioning. Molecular Ecology Resources. 2021; 21(2):609–620.

## Supporting information

**S1 Fig. Maximum Likelihood (ML) tree of freshwater species of Bangladesh based on nucleotide sequences of COI gene.**

**S1 Table. Uncorrected *P* distance among the analyzed taxa**

**S2 Table. Result of species delimitation analyses**

**S3 Table. Possible cryptic species found in this study**

Click here to access/download;Figure;Fig. 1.eps

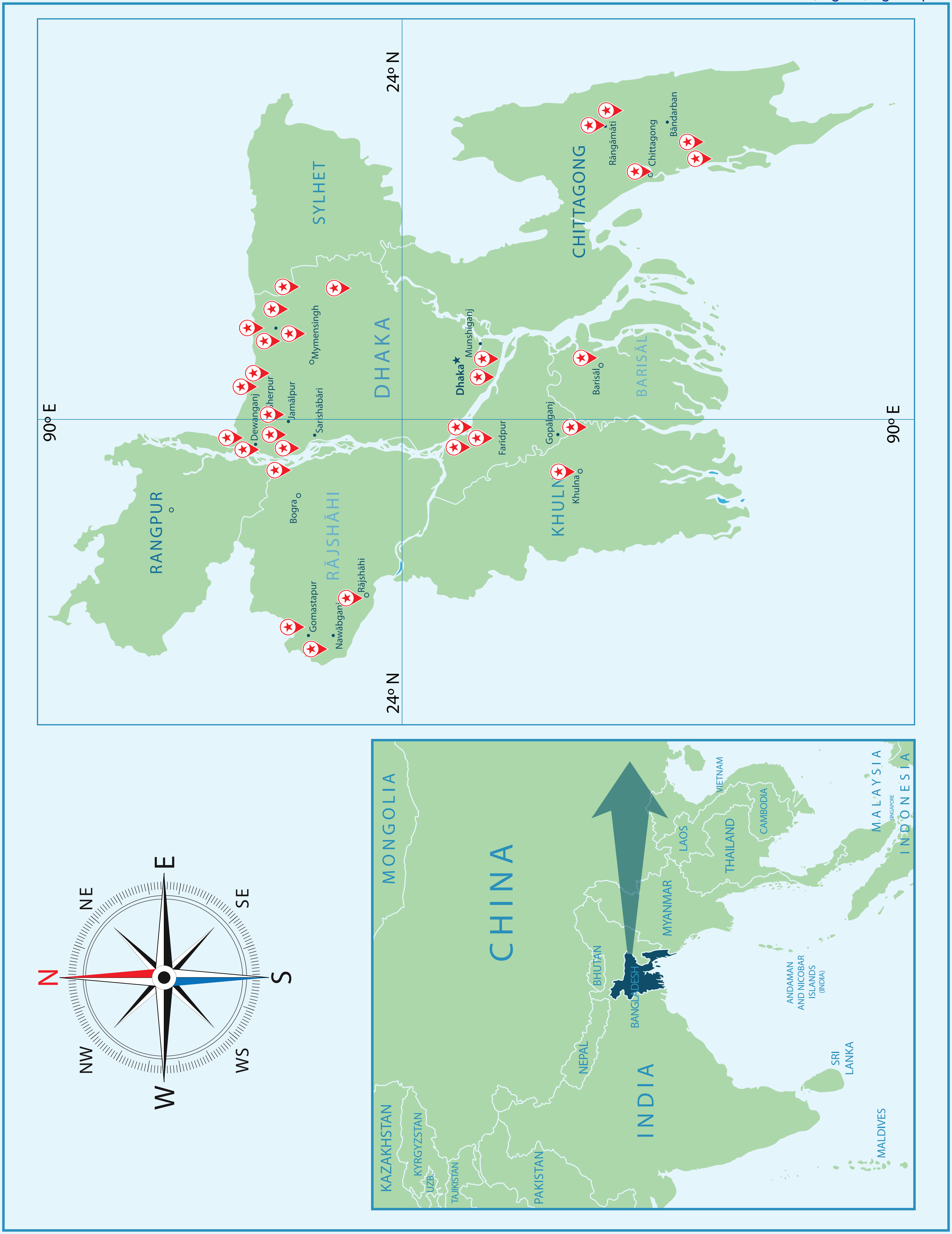

Click here to access/download;Figure;Fig. 2A.tif

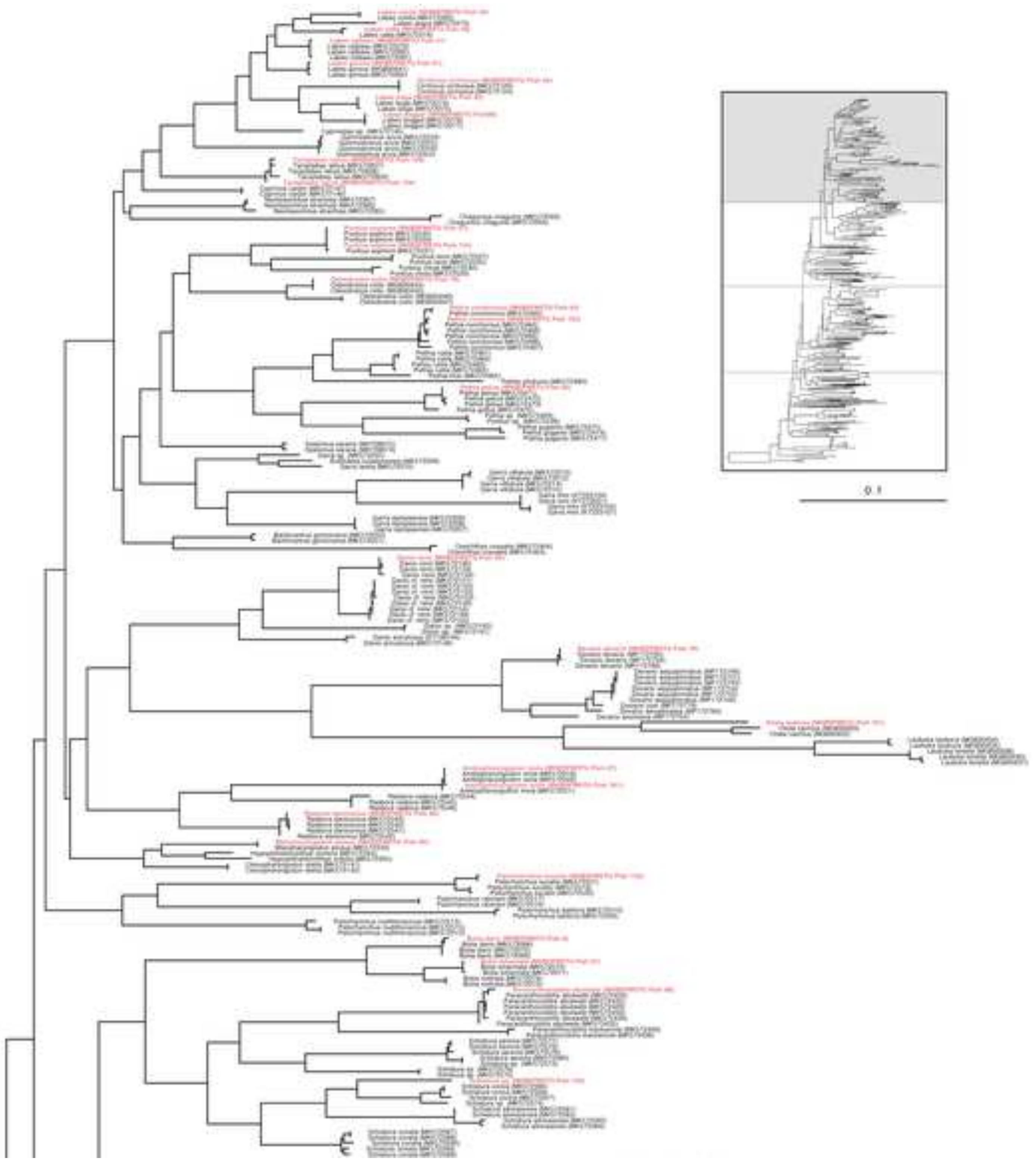

Click here to access/download;Figure;Fig. 2B.tif

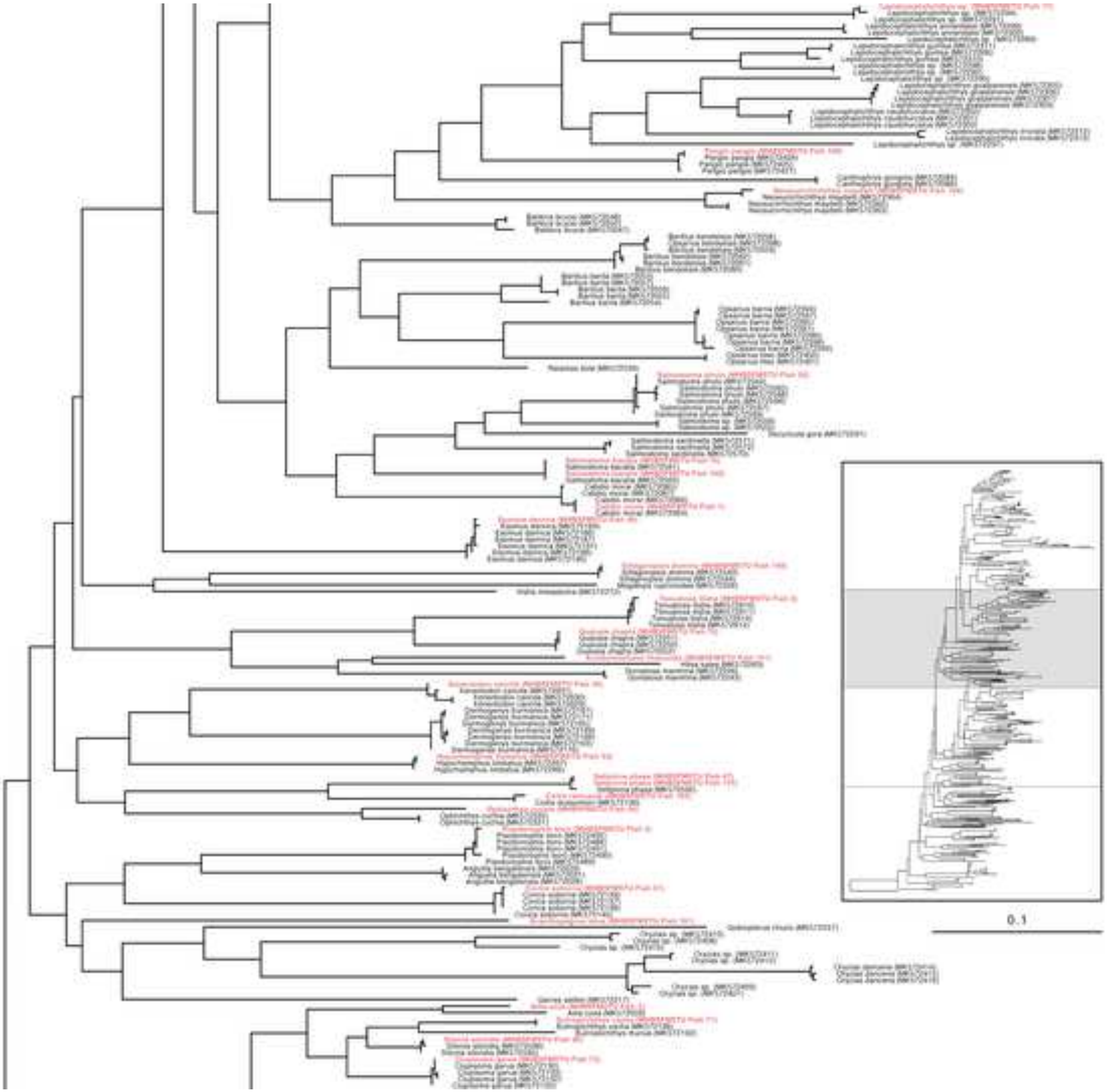

Click here to access/download;Figure;Fig. 2C.tif

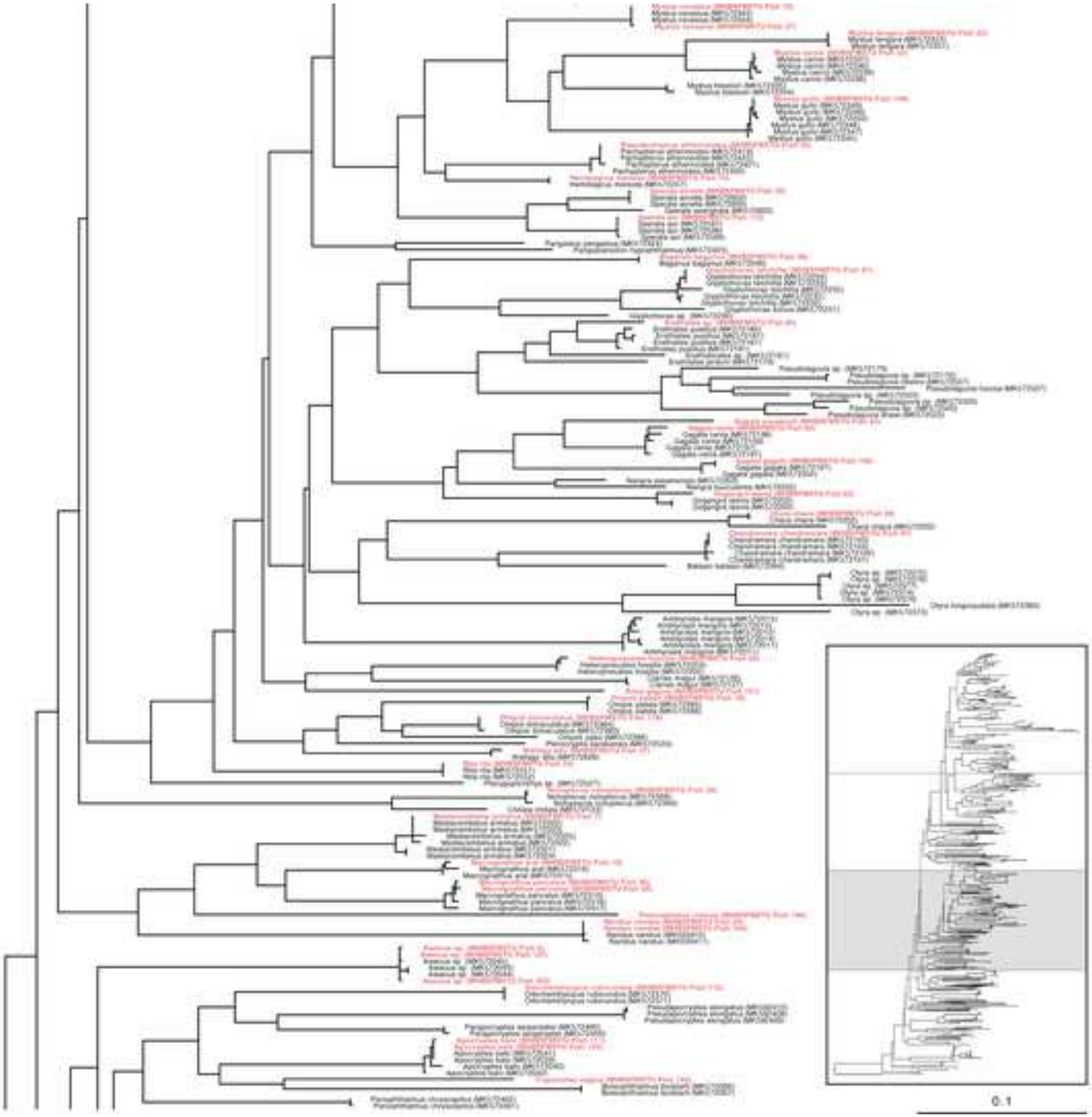

Click here to access/download;Figure;Fig. 2D.tif

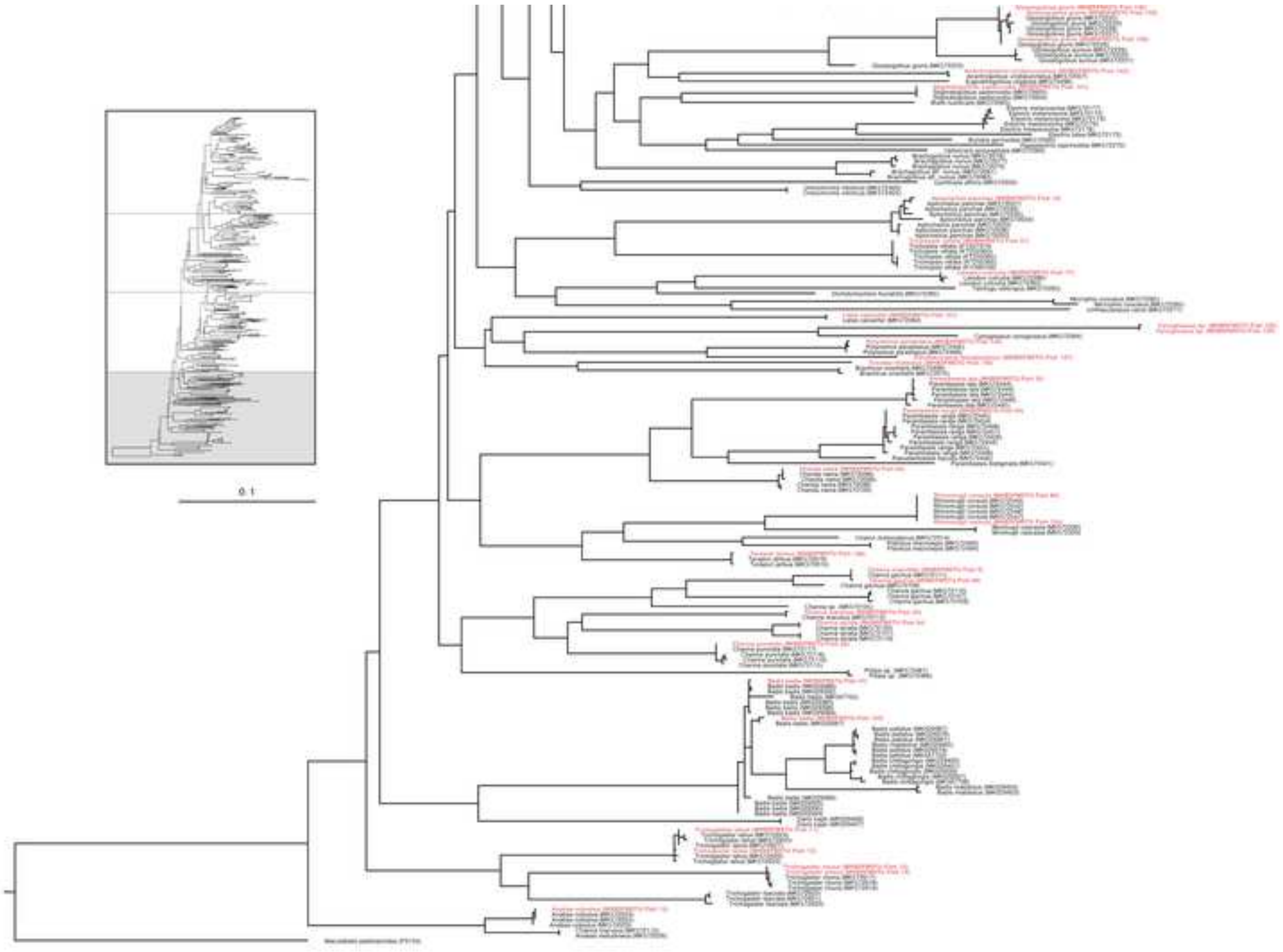

Click here to access/download
**Supporting Information**
S 1 _Table.csv

Click here to access/download
**Supporting Information**
S 2 _Table.txt

Click here to access/download

**Supporting Information**

S 3 _Table.docx

Click here to access/download
**Supporting Information**
S1_ Fig..tre
