# Supplementary material for: Cryptic biodiversity of freshwater fish species in Bangladesh
Source: PLoS One. 2025 Apr 28;20(4):e0318982. doi: 10.1371/journal.pone.0318982 (PMC12036941; doi:10.1371/journal.pone.0318982)
Supplement: Attachment — Submitted filename: Response to Reviewers-PONE-D-24-38143-Hasan et al..doc [file pone.0318982.s009.doc]

**PONE-D-24-38143**

**Cryptic biodiversity of freshwater fish species in Bangladesh**

**PLOS ONE**

**Reply to Academic Editor:**

We sincerely appreciate your useful comments and recommendations. These comments and recommendations have greatly aided us in revising our manuscript. We have edited the manuscript in accordance with your recommendations. We have also responded to each of your individual remarks as below (all page(s) and line(s) number mentioned here based on the clean view file):

1) Your comment: Please provide the family name for each species following the catalog of fishes.

*Our Answer: We provided the family name for each species following the catalog of fishes. Please see Table 1 in Pages 4-7.*

2. Your comment: Abstract & Introduction: need to check English texts for spelling, grammar, punctuation, and space.

*Our Answer: We resurrected and improved Abstract & Introduction section relating to spelling, grammar, punctuation, and space. Please see in Pages 1-3.*

3. Your comment: Materials: why the authors didn't conduct morphological analyses or morphometric analyses, which are essential in cryptic species analyses. I believe the authors should examine the most important morphological characters of fish species to identify if there are any significant differences. I suggest the authors investigate this aspect further in their study.

*Our Answer: Initially, our objective was to identify potential cryptic species using the DNA barcoding method (COI gene). During our revisions and based on your comment, we integrated morphological parameters with images of potential cryptic species. The initial evaluation included a limited morphological observation of these cryptic taxa. Refer to Table 2 on Pages 10-12. In future, we will examine details morphological characteristics by comparing them with specimens from the original site for new species descriptions (Type locality).*

4. Table arrangements: Table one is not in a good shape. For example, please provide longitude and latitude values instead of the word GPS, to be more specific, about what the authors mean by GPS.

*Our Answer:* [*We rearranged the Table 1. We provided latitude and longitude instead of GPS. GPS means Global Positioning System which integrates the location. In Table 1, we deleted tissue collection date which does not decline the quality of the Table. Please see Table 1 in pages 4-7.*](https://www.gps.gov/)

[5. Results section: Need to create a new table that includes all the cryptic species for better clarity and harmony without providing the fish number in the text.](https://www.gps.gov/)

*Our Answer: We created a new table that includes all the cryptic species for better clarity and harmony without providing the fish number in the text. Please find it in pages Pages 10-12.* [*In*](https://www.gps.gov/) *our result section we used species name with family without mention the fish number. Please see result section thoroughly (Pages 9-11).*

6. Discussion: is poor written and has to be improved based on the obtained results and referencing previous studies for comparison.

*Our Answer: We improved the “Discussion” section by incorporating comparative previous studies with good referencing. Please see discussion section thoroughly (Pages 12-15, Lines 175-250).*

*Figure 1 was re-drawing and supplied the details information in the legend of Fig.1.* The map is constructed in ArcMap 10.8 software using free vector and raster data available by Natural Earth ([https://www.naturalearthdata.com](https://www.naturalearthdata.com/)). All maps are available in public domain (<https://www.naturalearthdata.com/about/terms-of-us>).

*Our genetic sequences data will be available after acceptance of the manuscript (Accession numbers LC823243–LC823366). Other relevant data are provided with supporting files (S1-Table, S2_Table and S1_Fig) with this manuscript.*

**Reply to Reviewers:**

We deeply appreciate for your valuable comments and suggestions. These are greatly helpful in revising our manuscript. Basically, we revised the manuscript according to your suggestions. We also reply to your specific comments as followings.

Reviewer #1: Your comment: the manuscript is very interesting, explaining a problem that is emerged and is getting more and more attention. Your manuscript has some problems regarding the existence of words that have been merged between them. Please check the text for correcting them. Also I have suggested some minor corrections to some parts of the text.

*Our answer: We appreciate your interest and attention to our MS. We are very grateful to you. We dispersed the fused words throughout the manuscript. We have also implemented minor revisions to our manuscript as outlined below:*

Your comment: You should mention here which features have been tested in this study. To make a clear view of the reader

*Our answer: Initially we tested COI gene data (DNA barcoding) and sorted out the possible presence of cryptic species. Later, we applied restricted morphological examination of these cryptic taxa (Page 1, Lines 20-21). Further investigation is warranted during the new species description including the topotypic specimen.*

Your comment: I am wondering here: why did the authors have used two chemical materials to preserve the taxa for the subsequent analyses? And why did they use 95%, not 70%, of ethanol?

*Our answer: For subsequent molecular research, the species were maintained in either saturated DMSO/NaCl solution or 95% ethanol. Because, specimens preserved in DMSO/NaCl solution does not affect any quality rather provide better quantity of DNA concentration. Alternatively, we also preserved our specimens in 95% ethanol instead of 70% ethanol for maintaining good quality for long time. Because, to start DNA work it takes around 3-4 years from our sample collecting date.*

Your Comment: I suggest using longitude and latitude instead of the word GPS, to be more specific, about what do the authors mean by GPS.

*Our Answer:* [*We rearranged the Table 1. We provided latitude and longitude instead of GPS. GPS means Global Positioning System which integrates the location. In Table 1, we deleted tissue collection date which does not decline the quality of the Table 1. Please see Table 1 in pages 4-7.*](https://www.gps.gov/)

[Your Comment: I'm still wondering why the authors didn't conduct morphological analyses or morphometric analyses, which are essential in cryptic species analyses. I believe the authors should examine the most important morphological characters of fish species to identify if there are any significant differences. I suggest the authors investigate this aspect further in their study.](https://www.gps.gov/)

*Our Answer: Initially, our objective was to identify potential cryptic species using the DNA barcoding method (COI gene). During our revisions and based on your comment, we integrated morphological parameters with images of potential cryptic species. The initial evaluation included a limited morphological observation of these cryptic taxa. Refer to Table 2 on Pages 10-12. In future species descriptions, we will examine details morphological characteristics by comparing them with specimens from the original site (Type locality).*

Your comment: I have not seen any morphological results in this manuscript.

*Our Answer: We provided limited morphological characters with the morphometric ratios (Please see Table 2). It is just preliminary result and observation. In future, we will deeply investigate this issue.*

Your comment: Could you mention here what the value of the threshold genetic distance that has been used to prove these seven taxa are new species? And cite here the previous studies that have suggested that this gnetic distance can be used to separate species as new species, not lineages.

*Our Answer: First of all, 7 taxa are not new species rather they are possible cryptic species which might be new species. To confirm their new species identity, further molecular, deep morphological analyses required. In this work, intraspecific genetic divergence exceeds the minimum intraspecific distance of 3% (the COI gene), which is higher than the threshold value of 2% for fish species identification via DNA barcoding .Generally closely related vertebrate species consistently demonstrate over 2% divergence in the mitochondrial cytochrome b gene and COX1 gene for conspecific genetic divergence in fish. Fouquet et al. demonstrated that a 3% cutoff value for 16S mtDNA yielded a greater number of potential neotropical frog species. Please see pages 14 and lines 213-218.*

Your Comment: Why do the river systems play important role in the process of speciation?

*Our answer: Despite being a low-lying country, Bangladesh’s robust river systems impede gene flow and affect the population structure of Hoplobatarchus tigerinus frog (See our previous paper ref no. 30).  Further, both intrinsic species characteristics, such as dispersal capability and habitat specialization, and extrinsic environmental factors, such as physical obstructions and geographic separation, often determine the level of population connectivity and gene flow in the freshwater ray-finned fish Etheostoma lemniscatu. Please see with ref page 13 lines 195-200.*

Your comment: This current study has not used integrative taxonomy.

Our abswer: Yes, this study lack of integrative taxonomy study. We re-write the Conclusion section. Please see page 15, lines 255-261.

**Reviewer #2: Dear Authors**

I had a very close look at the MS submitted to PlosOne journal and found it interesting. However, before it be considered for publication several issues should be fixed.

1) Your comment: Please use those keywords which have not been given in the title:

Ichthyodiversity, DNA barcoding, Molecular taxonomy

*Our answer: We used those keywords which have not been given in the title. Please see Page 2, Line 30.*

2) Your comment: fish base is not updated data base and it is usually used for commercial or fisheries purpose. The main acceptable data base is catalog of fishes. You have to check the valid species name using this database:

<https://researcharchive.calacademy.org/research/ichthyology/catalog/fishcatmain.asp>

*Our answer: According to your suggestion, we updated the family name and other fisheries information based on catalog of fishes.*

3) Your comment: Please provide the family name for each species following catalog of fishes.

*Our answer: Please see the table 1 where all family name was incorporated following catalog of fishes.*

4) Your comment: Table one is not in a good shape.

*Our answer: We improved the Table 1. Please see pages 4-7.*

5) Your comment: in the result section, no need to provide fish number in the text. I suggest to give all the cryptic species in a new table and provide significant findings in the result section.

*Our answer: We insert a Table 2 incorporating data of cryptic species. In connecting this, we provide relevant information in result section. Please see P.10-12.*

6) Your comment: Discussion is poor and has to be improved based on the obtained results and comparison with other related studies.

*Our answer: We improved the discussion and compared our results with other related studies. We improved the “Discussion” section by incorporating comparative previous studies with good referencing. Please see discussion section thoroughly (Pages 12-15, Lines176-250).*

7) Your comment: No conservation management text is given based on the obtained results.

*Our answer: We added few sentences about conservation management. Please find it P. 15 and L. 245-250.*

8) Your comment: References should be double-checked in the main text and reference section following journal's format strictly.

*Our answer: We added 14 new references and checked the main text with reference style. It seems appropriate. Please see ref section in P. 17-22.*

9) Your comment: The introduction should be focused on the main and significant purpose of the ms and providing some new and update information on the cryptic species and DNA barcoding.

*Our answer: We improved the introduction part which align with the main and significant purpose of the ms. We also provided some new and update information on the cryptic species and DNA barcoding both in introduction and discussion section. Please see P. 2 and L. 48-49 and P. 13-15.*

10) Your comment: No morphological data of cryptic species has been provided. Photos of them are needed.

*Our answer: We incorporated morphological data of cryptic species in Table 2. We also added photos of these species. But qualities of photos are not good in shape. Sometimes fin, scale and other parts of fish body have been distorted. It is quite difficult to explain all the morphological parameters without observing the topotypic specimens in this state.*

11) Your comment: I suggest following the attached article published on the same subject in PlosOne. It helps to improve the ms

*Our answer: We studied your recent publication and get some new knowledge. We applied this information in our MS. Please see L.405 and ref. 39.*

More are given in the attached pdf file of the Ms.

Your comment: it is about 30.2% (Fricke et al., 2024) catalog of fishes:

https://researcharchive.calacademy.org/research/ichthyology/catalog/SpeciesByFamily.asp

*Our answer: We used this information based on catalog of fishes. Pls see P. 2, L.42.*

Your comment: not much related. please delete it

*Our answer: We kept fishing……..culture and nutrition. The remaining part was deleted.*

Your comment: fishbase is not updated .The main acceptable data base is catalog of fishes.

You have to check the valid species name using this database: <https://researcharchive.calacademy.org/research/ichthyology/catalog/fishcatmain.asp>

*Our answer:* *According to your suggestion, we updated the family name and other fisheries information based on catalog of fishes.*

Your comment: all the species names should be checked using catalog of fishes

Please add the family name of each species

*Our answer: We improved all the species names based on catalog of fishes*

Your comment: no need to provide fish number here in the text. I suggest to give all the cryptic species in a new table.

*Our answer: We deleted all fish number from the MS particularly result and discussion section.*

Your comment: conservation management has not been discussed.

*Our answer: We discussed conservation issue shortly. Please see P. 15, L; 245-250*

Your comment: should be double checked following journal's format

*Our answer: The Ref section has been checked and improved which has the consistent with the journal style and format.*

Reviewer #3:

Your comment: The MS addresses the biodiversity of fish in Bangladesh, particularly intending to show that what is known today is greatly underestimated due to the methodologies used for past sampling and inventories. Although the MS brings interesting news, it does not seem to me that the sampling carried out by the authors is sufficient for an adequate analysis of the country's diversity (one of the objectives of the research), given the size of the country's drainage network and the number of specimens collected.

*Our answer: Yes, the MS is partially addressing the fisheries biodiversity of the country. Our main focus was to find out the cryptic/candidate species. Our specimen collection areas covered all the part of the country.*

Your comment: In addition, the authors should carry out a good review of the text, correcting several cases of joined words, particularly involving species names, and improving the writing style.

*Our answer: We revised the whole MS including good review. We also resurrected the joined words, species name and writing style.* We added new 14 related literatures in this revision.

Your comment: Furthermore, the results should clearly show the number of orders, families and species sampled and how much the survey carried out can represent in relation to the real diversity (there are no estimates that can evaluate the collection effort carried out); it is also very important to compare the results obtained with previous ones, mainly Rahman (2005) and Rahman et al. (2019).

*Our answer: We incorporated the number of orders, families and species sampled in the result section. We also compared our data with Rahman (2005) and Rahman et al. (2019).* *There are 265 freshwater species belongs to 164 genera and 57 families presence in Bangladesh (Rahman 2005) and Rahman et al., 2019 collected 694 specimens from all over the country which represent 243 species level Operational Barcode Units (OBUs). However in our study we able to collect 121 species (*94 genera, 39 families, and 19 orders*) which seems that the samples number of present study is limited. While undertaking collection of samples, we confronted with many difficulties particularly the non-availability of the targeted specimens in our water bodies. The current number of samples fairly extensive and includes most of the available species, however still is incomplete, we assume.*

Your comment: Furthermore, the authors fail to fulfill their objective "to review the biodiversity of Bangladesh's fisheries"; however, they show that the numbers may be underestimated due to the tools and techniques used previously, which were inefficient in identifying the existence of cryptic species.

*Our answer: Our main focus was to explore and clarify the cryptic/candidate species. Although we partly discuss the fisheries biodiversity but decline to calculate statistical value/richness of biodiversity.*

Your comment: In this sense, I recommend that the ms be rejected in its current form, but I encourage its resubmission in a format that incorporates the suggestions presented here.

*Our answer: We revised the whole MS based on your comment which improved the merit of the MS. In the current state of the paper, it seems that the MS is quite eligible to publish PLOS ONE journal.*

Your comment in the attached pdf file of the Ms.

Your comment: I would suggest the authors to use the word "macroscopical"

*Our answer: We used the word "macroscopical” instead of “naked eye” in introduction section. Please see P. 2, L. 49.*

*M& M section the correction has been done as per your instruction. Please see P. 3; L. 70.*
